# Rapid Colonisation of Synanthropic Stone Martens in a Highly Urbanised Region: Insights From Temporal and Spatial Analysis

**DOI:** 10.1002/ece3.71392

**Published:** 2025-05-26

**Authors:** Karen Cox, Jan Gouwy, Joachim Mergeay, Sabrina Neyrinck, Koen Van Den Berge

**Affiliations:** ^1^ Research Institute for Nature and Forest (INBO) Geraardsbergen Belgium

**Keywords:** GBS, landscape genomics, *Martes foina*, recolonisation, temporal

## Abstract

Medium‐sized carnivores, including the synanthropic stone marten ( *Martes foina* Erxleben, 1777), have shown remarkable adaptability to urbanised and fragmented landscapes, facilitating their spread across mainland Europe. This study investigates the recolonisation of a highly urbanised region by stone martens within two decades, examining spatial and temporal genome‐wide data (using genotyping by sequencing) to reveal colonisation dynamics, sources, and barriers influencing their expansion. Using genotypes from 5536 SNPs across 376 stone martens collected between 1995 and 2013, our findings indicate that stone martens successfully expanded through urban environments, yet dispersal was neither entirely random nor strictly distance‐dependent. The initial southeastern stronghold (E1) showed the lowest genetic diversity and limited spatial expansion, while other population sources contributed to recolonisation, highlighting a complex, multi‐source expansion. Gene flow in the early stages was largely confined to E1, progressing northward and eventually enabling exchange with a second eastern lineage (E2). Meanwhile, the western lineage displayed higher connectivity, occasionally crossing barriers like motorways. Motorways, however, significantly shaped recolonisation patterns, reducing gene flow, while other elements such as built‐up areas, secondary roads or waterways showed an additional though very small effect. Over the study period, genetic patch size increased, indicating longer dispersal distances. Gene flow strengthened within both eastern (E1 and E2) and western populations. Still, the western population diverged into two subclusters (W1 and W2) of which one became more differentiated. This suggests limited genetic homogenisation in the near future. This study provides insights into the genetic and ecological dynamics of carnivore recolonisation in highly fragmented landscapes.

## Graphical abstract

This study investigates the recolonisation of a highly urbanised region by stone martens within two decades, examining spatial and temporal genome‐wide data (using genotyping by sequencing) to reveal colonisation dynamics, sources, and barriers influencing their expansion. Our findings indicate that stone martens successfully expanded through urban environments, yet dispersal was not entirely random nor strictly distance‐dependent. This study underscores the adaptive responses of stone martens to urbanisation, providing insights into the genetic and ecological dynamics of carnivore recolonisation in fragmented landscapes.

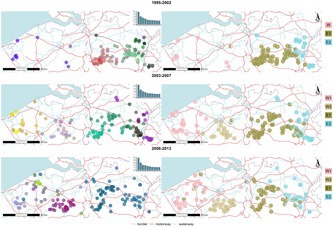

## 1 Introduction

Grasping how species are distributed and their abundance is vital for effective wildlife conservation and management. Land‐use changes can alter both aspects of a species. Habitat loss and agriculture are especially major drivers of biodiversity loss, posing significant threats to many terrestrial species (Tilman et al. 2017). Carnivores, in particular, are highly vulnerable to changes in the landscape caused by human activities (Ripari et al. 2022). Mammalian carnivores are among the most persecuted species and have undergone dramatic reductions in their geographic ranges (Ripple et al. 2014). Yet, they play a critical role in terrestrial ecosystems by controlling prey populations and affecting the entire food web (Estes et al. 2011; Soulé et al. 2003).

Medium‐sized carnivores are generally the most successful in highly fragmented and urbanised landscapes (Crooks 2002). The stone marten ( *Martes foina* Erxleben, 1777), a mustelid often referred to as the beech marten, is an example of such a carnivore that successfully inhabits a range of environments. Initially spreading with Neolithic human settlements from the Near East to southern Europe (Sommer and Benecke 2004), the species can now be found throughout mainland Europe except for the British Isles and the colder regions of Norway, Sweden, Finland and northern Russia (IUCN 2024). It inhabits various habitats, such as different types of rural areas (e.g., Czernik et al. 2016; Lachat Feller 1993; Wereszczuk et al. 2017), yet is a prime example of a mustelid that has adjusted to living in urban areas in Europe (Broekhuizen and Müskens 2000; Duduś et al. 2014; Herr et al. 2010; Tóth et al. 2009). While the related pine marten ( *Martes martes* Linnaeus, 1758) is typically more abundant in predominantly forested regions, the stone marten can also be found in the Mediterranean and high‐altitude forests of Spain, Italy, and the Alps (Virgós et al. 2000; Virgós and García 2002; Zub et al. 2018). Their flexibility allows them to inhabit fragmented landscapes and high‐altitude villages, demonstrating their capacity to thrive in diverse environments, including urban centres, which contributes to their abundance in central Europe.

Due to their wide distribution and large population, stone martens are listed as a species of ‘Least Concern’ on the European Red List (IUCN 2024). However, intense hunting and trapping in the early 1900s severely reduced their numbers. Hunting records from several countries, including Germany, Denmark, and Switzerland, show that stone marten populations began to recover between the 1950s and 1970s (Libois and Waechter 1991; Stubbe 1993). In recent decades, a significant and previously unexplained increase in stone marten populations has been observed in several central and western European countries (e.g., the Netherlands, Broekhuizen and Müskens 2004; Poland, Wereszczuk et al. 2017). This trend is consistent with earlier reports from the International Union for Conservation of Nature (IUCN), which indicated stable or growing stone marten populations in Europe. Among the suggested drivers of the expansion are climate warming, given its thermophilic nature, and an increase in human‐modified landscape (Wereszczuk et al. 2017). Notably, within less than two decades, stone martens have managed to recolonise Flanders (northern Belgium) extensively, starting from a near‐absence (Van Den Berge et al. 2002, 2012, 2022). As a consequence, the number of human‐wildlife conflicts has increased. The conflicts affecting humans mostly concern property damage, such as predation of poultry, car damage and denning in inhabited buildings (Herr 2008), while those affecting stone martens include collisions with vehicles and secondary rodenticide poisoning (Baert et al. 2015). In addition, urban infrastructure can provide other challenges: limited and patchily distributed resources of high quality food (Murray et al. 2019), constrained dispersal and gene flow, and other genetic effects (Holderegger and Di Giulio 2010; Miles et al. 2019; Tucker et al. 2018).

An important step in the colonisation process is the transition from arrival to establishment. In general, colonisation success in new or fragmented environments depends on a species' ability to maintain a suitable home range, as well as the flexibility of its spatial strategies in response to environmental pressures. For stone martens, home range sizes vary widely across different regions and even within the same location, from 15 to over 800 ha, depending on habitat availability and resource distribution (e.g., Genovesi et al. 1997; Herr et al. 2009; Rödel and Stubbe 2006; Wereszczuk and Zalewski 2019). Notably, home range fidelity in stone martens is typically lower than in other marten species (Herrmann 1994; Larroque et al. 2018; Wereszczuk and Zalewski 2019). In solitary carnivores, female home range size depends on food availability, while male home range size and distribution are influenced by the spatial distribution of females (Santos and Santos‐Reis 2010). Males adjust their behaviour to maximise encounters with females and increase reproductive success (Sandell 1989; Zalewski et al. 2004). Dominant, usually larger and older males, adopt a roaming strategy, expanding their home ranges to encounter more breeding females. Conversely, subadult males establish smaller home ranges that overlap significantly with unrelated females' territories to improve their mating opportunities. These strategies result in significant variation in spacing patterns and home range sizes, which are dependent on local conditions (Genovesi et al. 1997). Behavioural flexibility may be an essential driver of colonisation in fragmented landscapes, enabling stone martens to optimise their reproductive success in human‐dominated regions.

**FIGURE 1:**
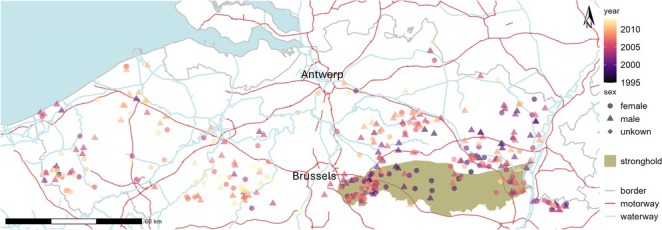
Study area in Flanders, northern Belgium, with country borders indicated with grey lines. Locations of the sampled stone martens are coloured according to sampling year and their shape depends on sex. The historical stronghold is shaded in green, the rivers, canals and the North Sea are in blue and the motorways in red. One sample in the Ardennes is not included; it is located 83 km south of the stronghold.

The recolonisation of northern Belgium (Flanders), a densely populated region, offers an opportunity to understand how habitat configuration and human‐induced landscape changes influence stone marten distribution. After their near disappearance in the early 20th century, stone marten populations began to rebound, likely shortly after World War II. However, until around 1998, this resurgence was largely confined to southeastern Flanders (Figure 1), with the species appearing only sporadically elsewhere. The rapid and substantial expansion in the following decades raised questions about the source populations and the factors facilitating their spread. Additionally, various aspects of the landscape matrix may have influenced the direction and extent of the colonisation. Previous genetic studies on stone marten (e.g., Vergara et al. 2015; Wereszczuk et al. 2017) have documented expansion patterns. However, they differ by temporal and/or spatial scale, as well as land cover configuration (e.g., level of urbanisation). To unravel the history of the recolonisation process in Flanders, we analysed samples collected across the study area between 1995 and 2013. The genetic characteristics, indicated by the extent of genetic diversity, the existence of non‐random spatial genetic structures, and the gene flow between different genetic clusters, are dependent on the population growth rates achieved by the initial founders and their descendants, as well as the movements that occur after colonisation events. Analysing genetic data in this context and in a temporal manner can provide valuable insights into the factors influencing the recolonisation of vacant habitats at both local and regional levels. Our investigation focused on three main questions: (1) Did the patterns of population structure change over time? (2) Could these patterns be explained solely by the effect of geographic distance on genetic variation? (3) Did land cover composition, particularly elements related to human activities, contribute to the observed changes in the population? By addressing these questions, we aim to shed light on the factors driving the resurgence and distribution of stone martens.

## 2 Material and Methods

### 2.1 Study Area and Sampling

The study was conducted in Flanders, the northern region of Belgium. This area, covering approximately 13,500 km^2^, is characterised by predominantly flat terrain. The landscape is dominated by vast expanses of arable land and pastures. The region is crisscrossed by a network of canals and waterways, roads and highways, and is highly urbanised. As such, it is among the most fragmented regions of Europe (Jaeger et al. 2011). Areas of woodland and forests are particularly present in the eastern part of the region.

Traffic casualties of small‐ and medium‐sized carnivores, including stone marten, were collected by a network of volunteers. At the 38 collection points and at the final destination (the Research Institute of Nature and Forest in Geraardsbergen, Belgium), the carcasses were stored at −20°C. We selected temporally and spatially distributed carcasses for sampling. A few samples were from the Brussels Capital region and one from the Ardennes in the Walloon region. The sampling period was from 1995 to 2013, with four samples dating from before the start of the network in 1998 (Figure 1). Due to limited processing and storage capacity, carcasses of stone marten were no longer collected systematically after 2013.

Stone martens become sexually mature between 12 and 28 months (Kollias and Fernandez‐Moran 2015; Larroque et al. 2016), can live between 5 and 10 years in the wild (The AnAge Database of Animal Ageing and Longevity, https://genomics.senescence.info), breed once a year and have an average litter size of around 3 young (Herr 2008; Kollias and Fernandez‐Moran 2015). Cementum age analysis was performed on teeth of a subset of 104 individuals (Matson's Laboratory, Milltown, Montana), sampled between 1995 and 2005, and showed that ages ranged between 1.5 months and 9 years (mean = 20 months, median = 12.5 months). Examination of reproductive characteristics of these and other individuals suggested a mixture of ages and reproductive states (unpublished).

### 2.2 DNA Extraction and Sequencing

In total, DNA from muscle tissue (mostly tongue or heart tissue) of 386 individuals was extracted using the DNeasy Blood & Tissue Kit (Qiagen) and eluted in 200 μL AE buffer. DNA of 50 extractions was diluted to a concentration of approximately 50 ng/μl, as measured using a Nanodrop2000 spectrophotometer (Isogen Life Science, Belgium), in a total volume of 10 μL. Partial restriction digestion was performed on those samples with the enzyme *Hind*III (Thermo Fisher Scientific) for a preliminary evaluation of the suitability of the DNA. The DNA template was digested with 1 U of the enzyme in a 20 μL reaction containing 2 μL of 10× assay buffer R (Thermo Fisher Scientific, USA) for 2 h at 37°C. Then, 10 μL of the digested products was analyzed on 1% agarose gel along with a Lambda DNA/*Hind*III size standard.

Genotyping‐by‐sequencing (GBS) was performed for constructing reduced representation libraries for the Illumina next‐generation platform. A *Pst*I GBS library of all samples, 83 replicated samples with one to three replicates each (18 replicates starting from tissue), and 5 blanks was prepared according to Elshire et al. (2011), and the resulting library was sequenced on an Illumina HiSeq 2000 (Cornell University Genomics Core Laboratory).

### 2.3 SNP Calling and Filtering

Trimming of Illumina adaptors and filtering of reads with a minimal length of 50 bp were performed using Cutadapt v4.2 (Martin 2011). Stacks v2.53 process_radtags was used to demultiplex raw reads and remove low‐quality reads (Catchen et al. 2013), followed by the removal of the restriction enzyme cut site with Cutadapt. We checked read quality using FastQC v0.11.9 after each processing step and using MultiQC v1.14 after the final step (Ewels et al. 2016). The software BWA v0.7.17 and more specifically the BWA‐MEM algorithm (Li 2013) was used for the alignment of the reads to the stone marten reference genome, the chromosome‐length (2n = 38) assembly available from the DNA Zoo Consortium (https://www.dnazoo.org/assemblies/Martes_foina, accessed on 8 September 2022; Dudchenko et al. 2017, 2018). After alignment, SAM files were converted to BAM files and sorted by position order using SAMtools v1.16.1 (Danecek et al. 2021).

Stacks was used to call SNPs with a reference genome (gstacks), allowing a maximum soft‐clipping level of 10% of the read length. The dataset was converted to VCF format for further analysis in RStudio v2022.12.0 + 353 (RStudio Team 2022) with R v4.2.0 (R Core Team 2022). Initially, we removed variants with a read depth of less than 3, a missingness of more than 30%, a minor allele frequency (MAF) of less than 0.01 or a minor allele count (MAC) of less than 3 using R package vcfR v1.13.0 (Knaus and Grünwald 2017). Paralogs were identified with the R script HDplot developed by McKinney et al. (2017) and removed. We further omitted potential paralogs or multicopy loci by removing variants with a read depth in the top 5% (i.e., with a read depth > 50). Next, samples with high missingness (≥ 80%) were removed.

Further filtering steps were performed using R package snpR v1.2.5 (Hemstrom and Jones 2023). Filtering thresholds were set to MAF > 0.01, a maximum observed heterozygosity of 0.55 and a missingness rate of less than 30% per locus. These filtering steps were repeated until all thresholds were reached. Subsequently, the same thresholds were applied, but with a missingness threshold per sample (< 50%), instead of per locus. SNPs were finally pruned for high levels of pairwise linkage disequilibrium (LD) when MAF > 0.01, missingness per sample < 30%, and a correlation coefficient existed of at least 0.5 between SNPs within a sliding window of a maximum 50 SNPs, using R package SNPRelate v1.28.0 (Zheng et al. 2012).

In order to filter SNPs that deviated from Hardy–Weinberg equilibrium (HWE), we first defined genetic clusters using principal component analysis (PCA) with R package adegenet v2.1.10 (Jombart 2008; Jombart and Ahmed 2011), with missing data imputed as the mean value per SNP. We grouped samples according to the PCA results, which showed five major genetic clusters. Using R package pegas v1.2 (Paradis 2010), we tested which SNPs deviated from HWE (*p* < 0.05). Those SNPs that tested positive in all five clusters were removed. SNPs potentially under selection were detected with pcadapt v4.3.3 (Privé et al. 2020) using five principal components and a correction for multiple testing by Benjamini and Hochberg (1995). The method implemented in R package OutFLANK v0.2 (Whitlock and Lotterhos 2015) was used as a second approach with samples clustered in the same five genetic clusters (see earlier). File conversions were performed with R package radiator v1.2.2 (Gosselin 2020).

### 2.4 Spatial and Temporal Population Structure

Population structure analysis was performed on all data using two methods: PCA as mentioned earlier and Discriminant Analysis of Principal Components (DAPC) (Jombart et al. 2010). For the latter approach, we first searched for clusters de novo by using the K‐means procedure with information from 19 principal components, with K numbers of clusters going from 1 to 20, and with 10 replicates per K. The best clustering model was selected based on the Bayesian information criterion (BIC). We used *K* − 1 leading PC axes as predictors of among‐population differences as suggested by Thia (2023). A third approach to assess population structure, but with estimation of individual admixture coefficients using sparse Non‐Negative Matrix Factorization algorithms (sNMF), was performed using the R package LEA v3.6.0 (Frichot and François 2015). This method calculates least‐squares estimates of ancestry proportions and ancestral allelic frequencies. It then computes the entropy criterion to evaluate model fit using cross‐validation. This is a tool to help choose the number of ancestral populations, which is linked to the number of principal components that explain variation in the genomic data. We used the following settings: 10 repetitions for each *K*, with *K* = 1–10, and the regularisation parameter values 10, 100, 500 and 1000.

For the following spatial analysis, we excluded the sample from the Ardennes, which is located outside the focal study area. The isolation‐by‐distance (IBD) pattern was tested using a linear mixed model with maximum‐likelihood population effects (MLPE) (Clarke et al. 2002) that takes the non‐independence of pairwise distances into account through the use of a residual covariance structure. We used the R package nlme v3.1‐162 (Pinheiro et al. 2023) to fit Euclidean genetic distances calculated with adegenet, and the correlation structure using R package corMLPE v0.0.3 (Pope 2021), using Euclidean geographic distances as the predictor variable. A likelihood‐ratio based, adjusted pseudo‐*R* ^2^ based on an improvement from the intercept‐only model to the fitted model was calculated with the R package MuMIn v1.47.5 (Bartoń 2023). Finally, spatial autocorrelation was investigated for each sex with SPAGeDi v1.5 (Hardy and Vekemans 2002). We calculated Moran's *I* among pairs of individuals within distance classes from 0 to 32 km in intervals of 4 km, from 32 to 40 km, from 40 to 80 km in intervals of 10 km, from 80 to 100 km, from 100 to 150 km, from 150 to 200 km, and from 200 to 240 km. Significant deviation of spatial autocorrelation from a random distribution of genotypes was tested with 1000 random permutations of individual locations for each distance class.

To investigate the evolution of population structure across the sampling period, we divided the samples into three temporal groups based on sampling year: 1995–2002, 2003–2007 and 2008–2013 (Table 1). Population structure for each period was assessed using PCA and sNMF. Genetic differentiation calculated as pairwise *F*_ST_ (Weir and Cockerham 1984) was estimated between the genetic clusters of each temporal set using R package diversity v1.9.90 (Keenan et al. 2013). Corrected 95% confidence intervals were calculated using 100 bootstrap iterations. In addition, the analyses of IBD and spatial autocorrelation (including standard errors for estimates by jackknifing loci) were performed for the three periods.

**TABLE 1:** Number of samples of stone marten in each of three periods, divided among genetic clusters according to sNMF results. The names of the clusters refer to those indicated in Figure 2. Population genetic statistics are given for each cluster in each period.

Period	Estimate	Cluster W	Cluster E1	Cluster E2
1995–2002	*N*	9	67	22
	*H*_o_	0.239 (0.003)	0.222 (0.002)	0.232 (0.002)
	*H*_e_	0.261 (0.003)	0.251 (0.002)	0.263 (0.002)
	*F*_IS_	0.062 (0.006)	0.103 (0.003)	0.098 (0.004)
	*A*_r_	1.916 (0.004)	1.774 (0.004)	1.793 (0.005)
	sMLH	0.996 (0.055)	0.994 (0.122)	0.997 (0.120)

Period	Estimate	Cluster W1	Cluster W2	Cluster E1	Cluster E2
2003–2007	*N*	21	21	71	31
	*H*_o_	0.232 (0.003)	0.242 (0.002)	0.219 (0.002)	0.204 (0.002)
	*H*_e_	0.251 (0.002)	0.261 (0.002)	0.245 (0.002)	0.252 (0.002)
	*F*_IS_	0.070 (0.004)	0.061 (0.004)	0.097 (0.003)	0.158 (0.003)
	*A*_r_	1.754 (0.005)	1.789 (0.005)	1.752 (0.005)	1.768 (0.005)
	sMLH	1.003 (0.140)	1.002 (0.108)	0.996 (0.125)	0.991 (0.166)
2008–2013	*N*	34	33	42	24
	*H*_o_	0.216 (0.002)	0.238 (0.002)	0.222 (0.002)	0.216 (0.002)
	*H*_e_	0.249 (0.002)	0.260 (0.002)	0.246 (0.002)	0.255 (0.002)
	*F*_IS_	0.117 (0.003)	0.078 (0.003)	0.082 (0.003)	0.137 (0.004)
	*A*_r_	1.760 (0.005)	1.779 (0.005)	1.750 (0.005)	1.777 (0.005)
	sMLH	0.994 (0.129)	0.999 (0.104)	0.997 (0.110)	0.996 (0.166)

Abbreviations: *A*_r_, allelic richness rarefied down to a sample size of 18 alleles (± SE); *F*_IS_, inbreeding coefficient (± SE); *H*_e_, expected heterozygosity (± SE); *H*_o_, observed heterozygosity (± SE); *N*, the number of individuals; sMLH, mean standardised multilocus heterozygosity (± SD).

### 2.5 Genetic Diversity and Relatedness

The following estimates of genetic diversity for each genetic cluster resulting from sNMF analyses were calculated using package hierfstat v0.5‐11 (Goudet and Jombart 2022): observed and expected heterozygosity (*H*_o_ and *H*_e_), allelic richness (*A*_r_) and the inbreeding coefficient *F*_IS_. Mean individual standardised multilocus heterozygosity (sMLH; Coltman et al. 1999) was computed with package inbreedR v0.3.3 (Stoffel et al. 2016) for every genetic cluster and each period. To further explore expansion patterns, we used generalised least‐squares models with the individual sMLH standardised by the mean heterozygosity across the entire dataset as the response variable, and longitude and latitude (as Lambert72 coordinates, centred and standardised) and their interaction as explanatory variables, with package nlme.

We used R package sGD v2.11 (Shirk 2015) to investigate patterns of genetic diversity (*H*_e_ and *A*_r_) within neighbourhoods across the three time periods. Wright (1946) introduced the concept of neighbourhood size (NS) in continuous populations which, assuming Gaussian dispersal, can be approximated by *π*(2*σ*)^2^ *D*, with *D* the density of effective individuals and *σ* the mean dispersal distance between parent and offspring. Within the genetic neighbourhood or breeding window with radius 2*σ*, mating can be considered largely random. We used the distance where the spatial autocorrelation drops to zero as the diameter. For the calculation of genetic diversity metrics, sGD uses package hierfstat. Estimates were generated when a neighbourhood contained at least nine samples. Using inverse distance weighting interpolation, with R package gstat v2.1.3 (Gräler et al. 2016; E. J. Pebesma 2004), estimates of genetic diversity were interpolated over the study area based on a 5 km grid created with R package stars v0.6.8 (Pebesma and Bivand 2023).

As a measure of movement, we calculated relatedness among individuals and visualised the relationships spatially. First, we tested multiple estimators, using the software Coancestry v1.0.1.9 (Wang 2011), on simulated genotypes with the same number of SNPs, allele frequencies and proportions of missing data as in our data. We chose a uniform allele drop‐out rate of 0.001 and an error rate of 0.05 per locus. For each of the following categories, 100 pairs of genotypes for dyads were simulated: parent‐offspring, full siblings, half‐siblings, first cousins and unrelated pairs. All available estimators included in Coancestry were evaluated: the moment‐based estimators of Wang (2002), Lynch and Ritland (1999), Ritland (1996), and Queller and Goodnight (1989), and the maximum likelihood estimators DyadML (Milligan 2003) and TrioML (Wang 2007). Pearson correlation coefficients between our estimated and expected relatedness values and the root‐mean‐square error (RMSE) within and across kinship categories were compared.

The best performing estimator on the simulated data was used to calculate relatedness (*r*) between all pairs of individuals of our empirical data, with an error rate of 0.05 per locus, using the software Coancestry. We subdivided the pairs into two major classes of relatedness using the lower limit of the 95% confidence interval of the simulated half siblings as a threshold. Pairs of individuals were considered highly related if *r* was above this threshold. They were considered moderately related if their *r* value was below the threshold but still greater than 0.0625, equivalent to the relatedness of second cousins.

### 2.6 Landscape Genomics

We performed a resistance analysis based on the residuals obtained from the isolation‐by‐distance method. Using the ResDisMapper v1.0 R package (Qian et al. 2020) average residuals across the study area within grid cells were calculated, and their significance was obtained using 1000 permutations. We chose 37 grid cells in each row and 116 cells in each column of the raster map of resistance, which corresponds with a resolution of approximately 2 km. Through the mapping of interpolated scores, areas with significant positive and negative residuals can be identified, which correspond with areas of restricted and greater gene flow, respectively. We used Prevosti's genetic distance (Prevosti 1974; Prevosti et al. 1975) and tested both linear and non‐linear IBD trends. Based on the results of the spatial autocorrelation analysis, the geographical distance was identified when genetic correlation reaches zero. This distance was then used as a threshold for calculating the residuals of sample pairs.

Distance‐based Redundancy Analysis (dbRDA), equivalent to a multivariate version of multiple linear regression (Legendre and Anderson 1999; Legendre and Legendre 2012), was used to associate genetic distance to different land cover types. This and other ordination methods are known for their ability to detect fine scale landscape genetic patterns (Balkenhol et al. 2009; Legendre and Fortin 2010). Linear models were built between the pairwise Bray‐Curtis distances among individuals linearised by a principal coordinates analysis (PCoA) and several explanatory variables. To represent the spatial structure in the genetic data, geographic distances among individuals were transformed into distance‐based Moran's eigenvector maps (MEM) using R package adespatial v0.3‐21 (Dray et al. 2023). The length of the longest edge of the minimum spanning tree was set as the threshold value for truncation of the geographic distance matrix. Only MEMs with positive eigenvalues were retained in the analysis, which account for main spatial patterns such as genetic structure (Dray et al. 2006).

Initially, the dbRDA was performed on the full model while partialling out the spatial effects (i.e., conditioning on MEMs associated to positive eigenvalues) using the vegan v2.6‐4 package (Oksanen et al. 2022). Statistical significance was assessed using 999 permutations and an adjusted *R* ^2^ ($R_{\mathrm{adj}}^{2}$) was calculated. Next, we applied the forward selection procedure to find the best model which maximises $R_{\mathrm{adj}}^{2}$ until $R_{\mathrm{adj}}^{2}$ of the full model was exceeded or a permutation *p* value of 0.05 for adding a term was exceeded. We used the variance inflation factor (VIF) to evaluate the presence of multicollinearity, with a threshold of 3.5 to remove variables before building the full model. We applied a variance partitioning analysis on the final models to disentangle the different landscape effects on genetic structure. The dbRDA was performed with the total dataset, the two regions east and west of Brussels separately, and for each period. Models for the eastern region included ancestry proportions as estimated with sNMF for one of the two genetic clusters as a covariate, thereby accounting for ancestral genetic structure.

Given the stone marten's adaptability to urban environments, potential reliance on tree cover for shelter, use of agricultural and semi‐natural areas for foraging, and vulnerability to road mortality we considered the following predictors: built‐up area, tree cover density, grassland cover, arable land cover, navigable waterways, motorways, primary and secondary roads. The first three land cover data were extracted from the European Union's Copernicus Land Monitoring Service information, where the following High Resolution Layers are made available: Impervious Built‐up Density 2018 (100 m; https://doi.org/10.2909/a807e528‐431a‐4dca‐a6cd‐0e8947563fce), Tree Cover Density 2012 (100 m, https://doi.org/10.2909/299ad2d6‐f2b8‐4716‐b169‐1d621250fc3c), Grassland 2015 (binary, 20 m, https://doi.org/10.2909/35a036bb‐c027‐401c‐8625‐2ecf722e8461). Arable land was extracted from CORINE Land Cover 2006 (100 m), provided by the same service (https://doi.org/10.2909/08560441‐2fd5‐4eb9‐bf4c‐9ef16725726a), which was reclassified using package terra v1.7‐29 (Hijmans 2023) to obtain a binary layer with two thematic classes (arable/non‐arable; with pastures as non‐arable). The following layers with navigable waterways were used: ‘Grootschalig Referentie Bestand’ (GRB) edition 2014 (Agentschap Informatie Vlaanderen), Hydrographic network of Brussels‐Capital Region created in 2002 and revised in 2015 (Bruxelles Environnement/Leefmilieu Brussel), Réseau hydrographique 1:10000 of the Walloon Region (accessed on 2022‐05‐11; Service Public de Wallonie), Vaarweg Informatie type vaarwegen of the Netherlands (version 2.0.0, accessed on 2022‐05‐11; Rijkswaterstaat), and OpenStreetMap data (www.OpenStreetMap.org) from Regierungsbezirk Düsseldorf and Kölhn in Germany (as of 2022‐05‐18 T20:21:26Z and 2022‐05‐11 T20:21:55Z, respectively), and from Nord‐Pas‐de‐Calais in France (as of 2022‐05‐17 T20:21:09Z). The same OpenStreetMap data from Germany and France, and data from Belgium (as of 2015‐03‐29) and the neighbouring provinces of the Netherlands (Limburg, Zeeland and Noord‐Brabant; as of 2022‐05‐11 T20:21:55Z) were used to extract data of motorways, primary and secondary roads. Projection transformations and filtering steps of the line features were performed with packages sf v1.0‐13 (E. Pebesma 2018; Pebesma and Bivand 2023) and tidyverse v2.0.0 (Wickham et al. 2019), respectively.

The proportion of the area surrounding the sampling locations covered by arable land, grassland, trees and built‐up, and the total length of navigable waterways, primary and secondary roads was derived by using buffers of different sizes. We used circular buffers with a radius of 2, 5, 10 and 30 km, representing several home ranges and dispersal distances recorded for this and related species (Herr et al. 2009; Vergara et al. 2015; Wereszczuk and Zalewski 2019), and accounting for the genetic patch size found using the spatial autocorrelation analysis. R package exactextractr v0.9.1 (Baston 2022) was used to extract the data within buffers from the raster layers, while package sf was used for the line features. The resulting variables were centred and standardised. The division of samples by motorways was used as a categorical variable (i.e., expanded to contrasts or dummy variables) and is therefore independent of any buffer radius. Both the dbRDA and variance partitioning were conducted separately for each buffer size and each of the three sampling periods (see earlier).

## 3 Results

### 3.1 SNP Filtering

One sample failed to sequence. After alignment, skipping primary alignments with insufficient mapping qualities (1.2%) and excessively soft‐clipped primary alignments (2.8%), 1,493,681 loci could be built that held 144,179 variant sites. After the first filtering steps using vcfR and HDplot, 5592 SNPs in 378 samples were retained. A further 56 SNPs were removed that showed deviations from HWE in all five genetic clusters. We found only four outliers using pcadapt, though no outliers with OutFLANK. Therefore, all SNPs were retained for further analysis. Two samples had uncertain metadata (location and year) and were, therefore, removed. In our final set, average missingness per SNP and per sample was 13.63%, and consisted of 376 samples (Table 1) and 5536 SNPs.

### 3.2 Spatial and Temporal Population Structure

The first four principal components explained most of the total variance (9%) in the total dataset. This corresponded with four spatial clusters (two clusters east of Brussels and two clusters west) (Figure S1). The *K*‐means results delivered no clear minimum in BIC, though an elbow in the curve could be found around *K* = 5 and 6 (Figure S2). Likewise, the cross‐entropy curve exhibited a plateau around 5 or 5–6 clusters, depending on the regularisation parameter *α* (Figure S3); we chose *α* = 100 showing minimal cross‐entropy values. Clustering results using DAPC and sNMF analyses were similar to the PCA results, yet showed a fifth cluster east of Brussels (Figure S4). Only when considering a sixth cluster did the results of DAPC differ from those of the sNMF approach; according to the DAPC, the majority of the stronghold formed a new cluster, while the sNMF added a cluster west of Brussels (Figure S4).

**FIGURE 2:**
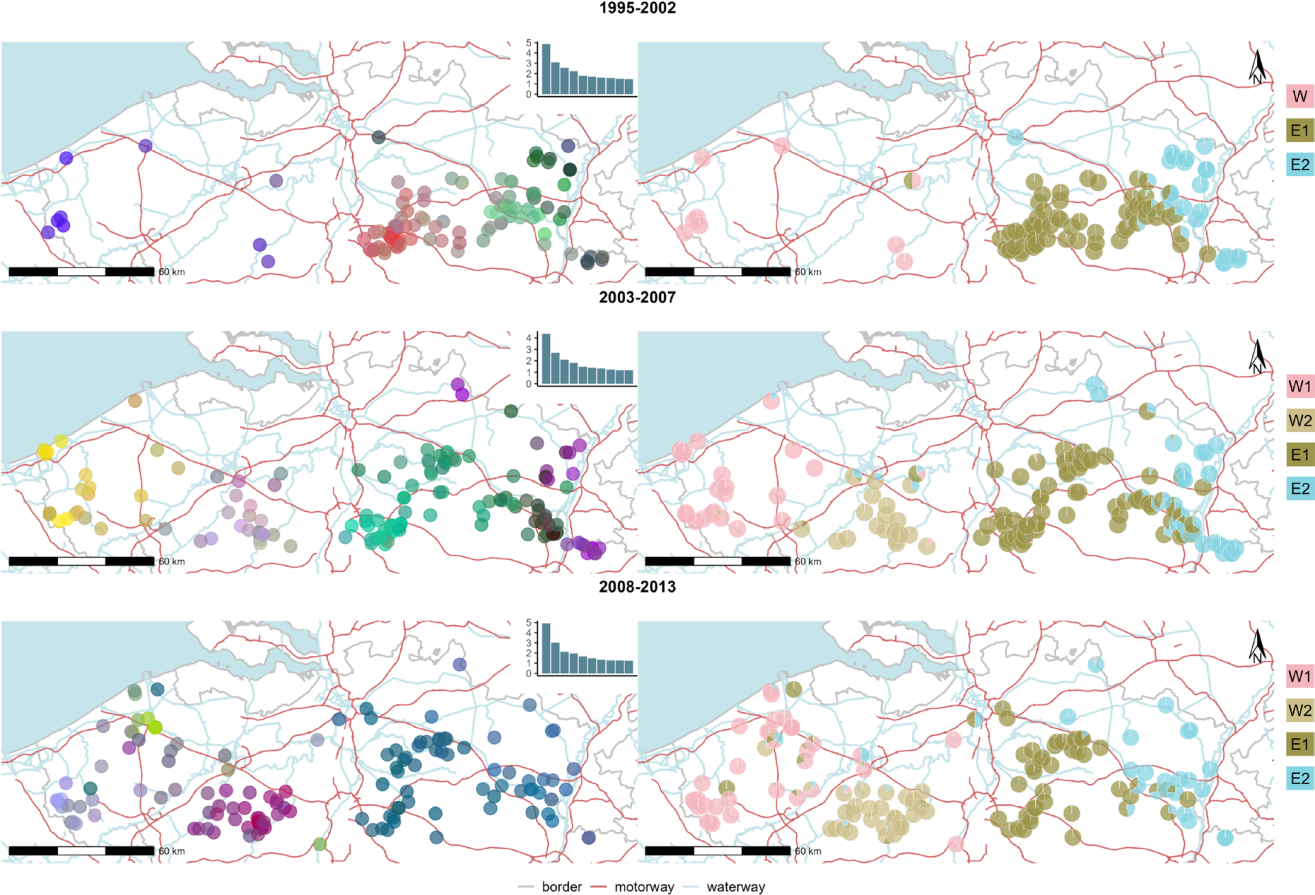
Genetic structure detected using PCA (left panel) and sNMF (right panel) for the three time periods, going from early (top) to most recent (bottom). Insets show the percentage of variance for the first 10 principal components. The PCA results are mapped for the first three principal components using coloured dots which summarise the values along the components assumed by each individual, using a gradient of the RGB colour system: Red (first PC), green (second PC), and blue (third PC). Cluster memberships obtained with the sNMF approach are shown as pie charts with a different colour for each cluster. For samples collected in 1995–2002 three genetic clusters were found, while for those collected in 2003–2007 and 2008–2013 four clusters were identified.

The first three axes of the PCA of the genetic data of 1995–2002 showed mainly three clusters with one that overlapped the old stronghold (cluster ‘E1’), one cluster more eastward (cluster ‘E2’) and a final cluster with the few individuals west of Brussels (cluster ‘W’, Figure 2). Similar results were found using the sNMF approach (*α* = 10; Figure 2). The following years, with increasing numbers of stone marten, these clusters east of Brussels maintained but showed more admixture in the contact zone, while two clusters formed west of Brussels, now called cluster ‘W1’ and ‘W2’ for reference. The sNMF results for *K* = 3 for the period 2003–2007 grouped clusters W1 and W2 together (results not shown). This suggests that they originated from the same ancestral cluster. The individual from the Ardennes (sampled in 2003 and located 83 km further south from the main study area) was assigned to cluster E2. In 2003–2007 and 2008–2013, individuals from eastern genetic clusters, or those with mixed eastern and western ancestry, were detected west of Brussels.

**FIGURE 3:**
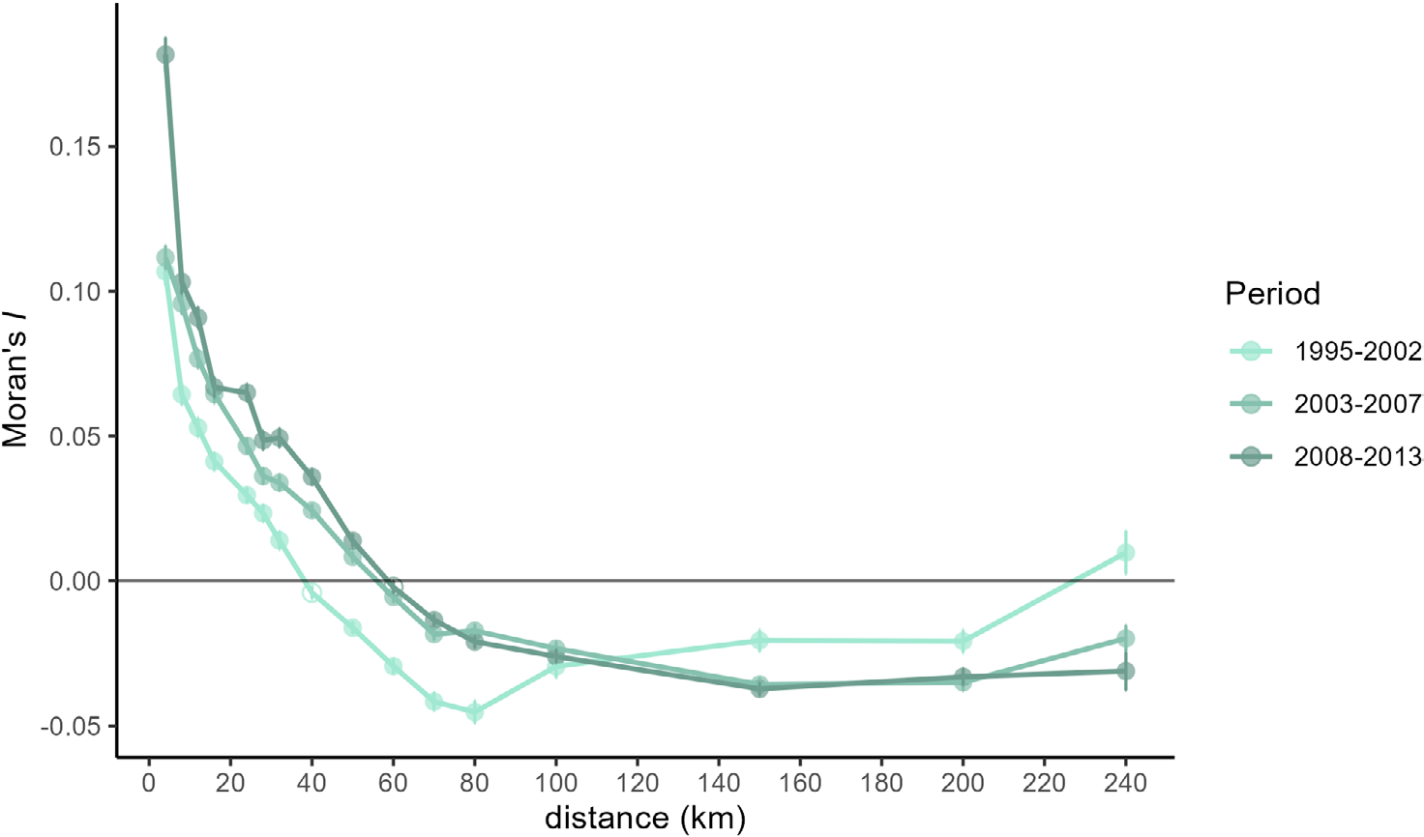
Moran's *I c*orrelogram for pairs of stone martens across 16 distance intervals. Each point represents the mean pair‐wise Moran's *I* with significance of spatial autocorrelation indicated as full circles and non‐significance as empty circles. Error bars delineate 95% confidence intervals from jackknife estimates.

In the IBD models for the total dataset and for each period separately, distance was highly significant (*p* < 0.001; Table S1), and the adjusted pseudo‐*R* ^2^ changed through time from 0.32 in 1995–2002 to 0.36 in 2001–2008 and finally to 0.39 in 2007–2013 (with $R_{\mathrm{adj}}^{2}$ = 0.38 for the total dataset). Spatial autocorrelograms were very similar for both sexes (Figure S5). Positive Moran's *I* increased over time and was significantly different from zero until a maximum distance of 32 km (mean = 30 km) was reached in 1995–2002, and of 50 km (mean = 45 km) in 2003–2007 and 2008–2013 (Figure 3).

Genetic differentiation among the genetic clusters east of Brussels, E1 and E2, decreased over time (Table S2). While pairwise *F*_ST_ decreased from the second to the third period between eastern clusters and cluster W1, the other western cluster, W2, became more differentiated from the east. In general, differentiation was highest between the eastern and western populations, which concurs with previous structure results.

### 3.3 Genetic Diversity and Relatedness

Heterozygosity, both *H*_e_ and *H*_o_, and *A*_r_ decreased slightly in the eastern genetic clusters (E1 and E2) from 1995–2002 to 2003–2007 (Table 1). Levels of genetic diversity in cluster E2 recovered partially in 2008–2013. Estimates for *H*_o_ and sMLH decreased in the western genetic clusters, while *A*_r_ slightly increased in cluster W1. In general, the least diversity was present in cluster E1, located in the area where stone martens never disappeared and its expansion front. The positive and sometimes increasing *F*_IS_ values could be due to the Wahlund effect when samples include an increasing amount of immigrants from other genetic clusters and due to admixture. While both latitude and longitude as well as their interaction were significant when all data was included in the model with sMLH as the response variable, only latitude appeared to be significant within the region west of Brussels (Table S3). The results suggest the highest genetic diversity is mostly located in the southwestern part of the study area, with its expansion front heading northwards.

**FIGURE 4:**
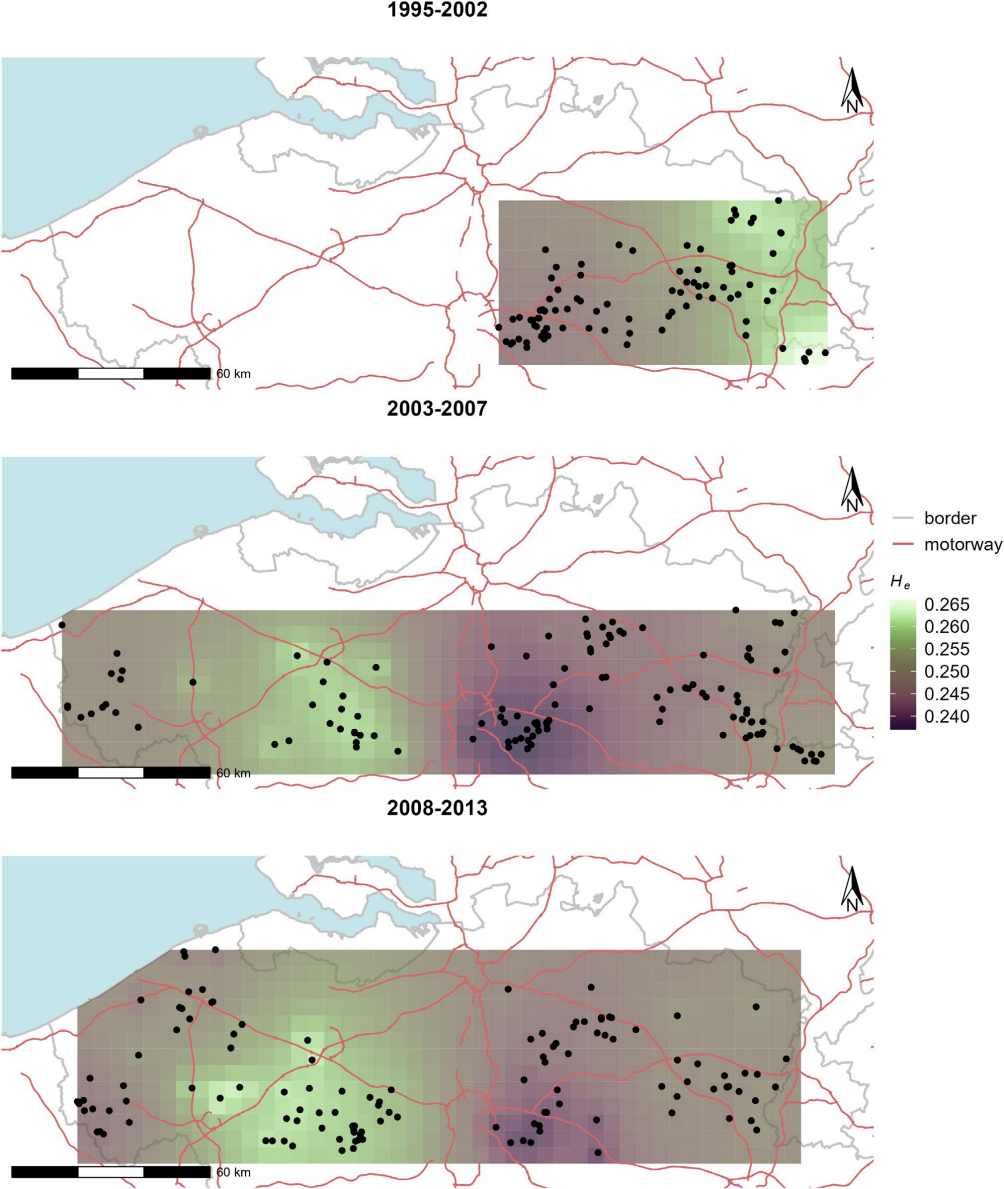
Spatial representation of expected heterozygosity (*H*_e_) within neighbourhoods with a radius of 25 km in periods 1995–2002, 2003–2007 and 2008–2013. Estimates at sampling locations were interpolated using inverse distance weighting and a 5 km grid. Grey lines are country borders and red lines major motorways.

Genetic diversity estimates were calculated for neighbourhoods using a diameter of 50 km. This distance approximated the distance where spatial autocorrelation reached zero (Figure 3, Figure S5). As *A*_r_ and *H*_e_ were highly correlated for each period, we visualised results for *H*_e_ (Figure 4). Results confirmed genetic diversity estimates per genetic cluster. The lowest values were found in the area where the stronghold and its cluster E1 reside, while the highest values were found in the area of individuals assigned to genetic cluster W2. A decrease in *H*_e_ in the region east of Brussels from the first to the second period is again apparent.

**FIGURE 5:**
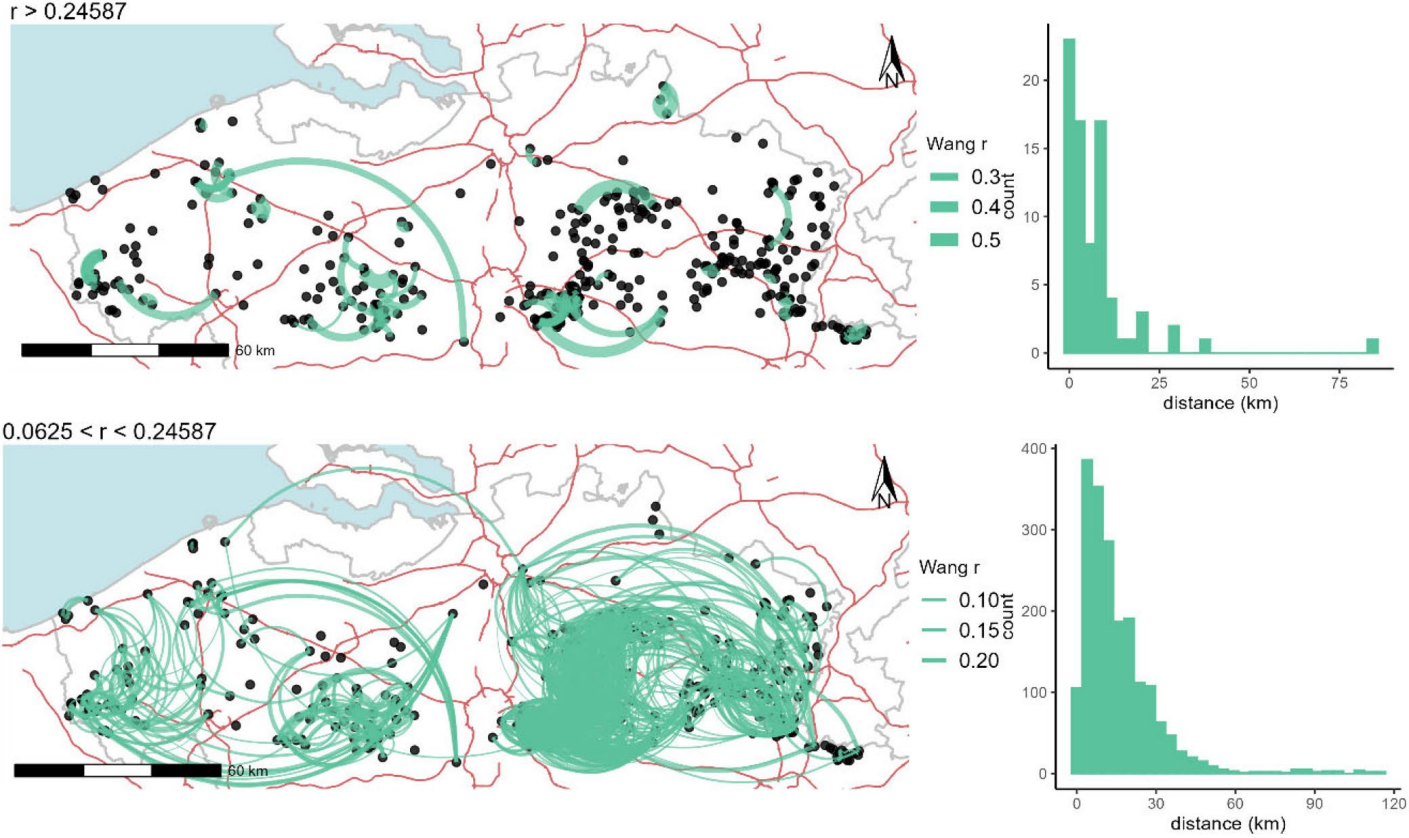
Wang's relatedness (*r*) between pairs of stone martens. Black dots indicate the locations of the sampled individuals. Highly related pairs with *r* > 0.24587 (the lower limit of the 95% confidence interval of the simulated half siblings), and pairs related to a lesser extent with *r* > 0.0625 (the equivalent of second cousins) are connected with green edges. Edge width increases with a higher *r*. The background shows the country borders (grey lines) and the motorways (red lines). Histograms of the distances for each of the two ranges of *r* are shown on the right.

Correlations between simulated and true relatedness values exceeded 0.99 for every estimator. Overall RMSE was the smallest for DyadML (0.0169) and TrioML (0.0171), while other estimators delivered RMSE between 0.0190 and 0.0207 with the exception of Ritland with an RMSE of 0.0276. Which estimator performed best within categories of kinship depended on the category. In general, estimator DyadML showed mostly the lowest or very close to the lowest RMSE, except for the full sibling relationship. As we were mostly interested in close kinships, we also chose the estimator of Wang, which performed better for parent‐offspring relationships (RMSE = 0.0190), and for full siblings (RMSE = 0.0190; RMSE = 0.0189 for Queller‐Goodnight). The lower limits of confidence intervals of simulated half siblings was 0.24156 for DyadML and 0.24587 for Wang. We compared kinship results using both estimators and found Wang to be more conservative in the number of (close) relatives found among the sampled individuals, and, therefore, only show these results (Figure 5). Of a total of 78 highly related dyads, 81% showed maximal distances of less than 10 km and 95% of less than 25 km. One dyad with *r* = 0.2861 was between a pair of individuals separated by a distance of 84 km. Less related pairs (0.0625 < *r* < 0.24587) were numerous (1951 pairs; Figure 5) with distances between 10 m and 115 km. Still, most of these dyads were within the regions east and west of Brussels. Only two dyads were found showing an east–west connection (with *r* = 0.0745 and *r* = 0.1327).

### 3.4 Landscape Genomics

Residuals of the IBD curve were calculated for pairs of individuals with a maximum distance of 50 km, the same distance used to approximate neighbourhoods, where Moran's *I* approached zero in the spatial correlogram (Figure 3, Figure S5). Results based on linear and non‐linear relationships between geographic and genetic distances were very similar, yet a non‐linear IBD trend showed a slightly better fit and was used for further analysis with ResDisMapper (Figure S6). As expected, at the early stages of expansion where most martens still resided in or near the stronghold, gene flow appeared to be confined to this region (Figure S7). Further in time, more northward migration in this region seemed possible, while gene flow further east was more restricted until 2008. Resistance to gene flow was high between the regions east and west of Brussels. In the most recent period, from 2008 to 2013, this separation remained largely intact, though with a corridor in the south. Due to unsampled regions south of our study area, this corridor south of Brussels predicted by ResDisMapper analysis could be inaccurate. Within both regions, east and west of Brussels, resistance to gene flow decreased over time.

While the proportion of arable land was correlated with other variables at all investigated buffer sizes, some other variables showed multicollinearity problems depending on the period and buffer size investigated. They were excluded from the full models before model selection (Table S4). Generally, a very small amount of variance was explained by the variables included. No model was built for samples collected in the western region during 1995–2002 because of their very limited number. Full models for this region were not significant for the period 2003–2007, and three models only slightly for the most recent period (Table S4). Model selection resulted mostly in intercept‐only models, except for two models with one including variable primary road density within a 2 km radius and one with tree cover density within a 10 km radius with $R_{\mathrm{adj}}^{2}$ = 0.01 (Table S5). When all samples of this region were included, a few variables remained, such as the density of navigable waterways, tree cover density and/or grassland cover, depending on the buffer radius. Yet, their effect was extremely small, with $R_{\mathrm{adj}}^{2}$ between 0.0049 and 0.0066 (Table 2). Overall, *p*‐values for the remaining variables in models for the western region were mostly just below the 5% level.

**TABLE 2:** Results of the distance‐based redundancy analysis (dbRDA) of genetic distances (Bray‐Curtis distances) and land cover properties conditioned on spatial structure (Moran Eigenvector Maps) for the total dataset, for the samples collected east of Brussels, and for the samples collected west of Brussels.

Set	Radius (km)	Model $R_{\mathrm{adj}}^{2}$	Variable	df	SS	*F*	*p*_term_	*p*_marginal_	$R_{\mathrm{adj}}^{2}$
Total	2	0.0102	Motorways	14	0.1331	1.2562	0.001	0.001	0.0102
	5	0.0112	Motorways	14	0.1331	1.2578	0.001	0.001	0.0103
			Navigable waterways	1	0.0103	1.3560	0.001	0.001	0.0011
	10	0.0113	Motorways	14	0.1331	1.2580	0.001	0.001	0.0101
			Navigable waterways	1	0.0093	1.2302	0.006	0.007	0.0007
			Tree cover density	1	0.0088	1.1593	0.042	0.035	0.0005
	30	0.0112	Built‐up	1	0.0110	1.4603	0.001	0.001	0.0099
			Motorways	14	0.1323	1.2499	0.001	0.001	0.0010
East	2	0.0294	Motorways	7	0.0758	1.4726	0.001	0.001	0.0124
			Ancestry	1	0.0252	3.4259	0.001	0.001	0.0098
			Grassland	1	0.0097	1.3149	0.005	0.001	0.0020
			Built‐up	1	0.0087	1.1878	0.026	0.011	0.0013
			Tree cover density	1	0.0092	1.2520	0.012	0.006	0.0012
	5	0.0307	Motorways	7	0.0758	1.4747	0.001	0.001	0.0102
			Ancestry	1	0.0252	3.4308	0.001	0.001	0.0107
			Navigable waterways	1	0.0090	1.2263	0.017	0.015	0.0012
			Grassland	1	0.0088	1.2005	0.016	0.028	0.0009
			Tree cover density	1	0.0086	1.1728	0.043	0.016	0.0010
			Sec. roads	1	0.0087	1.1857	0.029	0.006	0.0014
			Built‐up	1	0.0091	1.2407	0.014	0.016	0.0012
	10	0.0312	Motorways	7	0.0758	1.4755	0.001	0.001	0.0115
			Ancestry	1	0.0252	3.4326	0.001	0.001	0.0108
			Navigable waterways	1	0.0098	1.3373	0.001	0.012	0.0012
			Sec. roads	1	0.0099	1.3421	0.003	0.001	0.0020
			Tree cover density	1	0.0093	1.2671	0.005	0.017	0.0011
			Built‐up	1	0.0086	1.1771	0.042	0.036	0.0009
	30	0.0294	Motorways	7	0.0758	1.4725	0.001	0.001	0.0112
			Ancestry	1	0.0252	3.4257	0.001	0.001	0.0105
			Built‐up	1	0.0095	1.2977	0.003	0.008	0.0012
			Grassland	1	0.0090	1.2276	0.014	0.007	0.0013
			Tree cover density	1	0.0090	1.2180	0.021	0.014	0.0010
West	2	0.0066	Navigable waterways	1	0.0125	1.4666	0.010	0.012	0.0066
	5	0.0110	Tree cover density	1	0.0116	1.3750	0.023	0.012	0.0060
			Navigable waterways	1	0.0119	1.4130	0.020	0.036	0.0059
	10	0.0064	Tree cover density	1	0.0124	1.4532	0.009	0.011	0.0064
	30	0.0114	Grassland	1	0.0117	1.3778	0.022	0.019	0.0053
			Navigable waterways	1	0.0114	1.3416	0.041	0.034	0.0049

*Note:* Set indicates the dataset used for the analysis. The radius represents the buffer radius used to calculate the proportion of different land cover classes surrounding the sampling locations and total length of navigable waterways, primary and secondary roads. Motorways were included as a categorical (or dummy) variable, and ancestry as the proportion assigned using sNMF to one of the two genetic clusters in the eastern region. Model $R_{\mathrm{adj}}^{2}$ is the adjusted *R* ^2^ for the entire model, while the last column shows the value for each variable after variation partitioning.

Abbreviations: df, degrees of freedom; *F*, *F*‐statistic; *p*_marginal_, the *p*‐value of the marginal effect of the terms (each marginal term analysed in a model with all other variables); *p*_term_, *p*‐value per term (sequentially from first to last); SS, sum of squares.

All full models for the eastern region were significant, the 1995–2002 models only marginally significant (Table S4). While model selection of the 1995–2002 models resulted primarily in models with significant effects of the ancestral lineage, the other models also included motorways as a covariate (Figure 1, Table 2, Table S5). To a lesser extent, other variables such as built‐up area and density of secondary roads came into play as well. There was a small increase in the explained variance of the models from 1.7%–3.2% in the first two periods to 4.1%–5.4% in 2008–2013. In the latter period, the ancestral population became relatively less important in comparison to the influence of motorways in explaining genetic distance. As in the temporal models, the final models for the entire eastern dataset also included ancestry and motorways as significant variables in combination with secondary road density and/or grassland cover (Table 2). Furthermore, other variables such as navigable waterway density (5 and 10 km radius), built‐up cover and tree cover density remained after model selection, though with low $R_{\mathrm{adj}}^{2}$ values. Reduced models which included the total eastern dataset showed predominantly an effect of motorways and ancestry in every model, and of built‐up area at a buffer radius of 30 km (Table 2). Motorways also explained approximately 2% in the two most recent periods to 4% of the variance in 1995–2002. Additional variables associated with urbanisation (built‐up area, primary or secondary road density) explained 0.2 to 0.5% variance at the 30 km radius (Table S5). Overall, the effects seemed to be mainly associated with the division of the eastern and western region and structure within the eastern region.

## 4 Discussion

In this study, we investigated the rapid recolonisation of a highly urbanised region by the synanthropic stone marten. Our temporal and spatial sampling regime provided valuable insights into the direction and sources of this colonisation process. Our results indicate that stone martens experienced hindrance from the urban environment during their expansion. Expansion did not evolve in a panmictic manner or solely according to an isolation‐by‐distance pattern. Notably, the south‐eastern stronghold was not the only source population for this expansion and stayed largely intact, indicating a complex colonisation dynamic.

### 4.1 Temporal Spatial Structure and Source Populations

Analysis of population structure revealed a clear evolution of three lineages: one west of Brussels and two in the east. The eastern lineages included the old stronghold (cluster E1) and the lineage that was initially present near the eastern border of Belgium (i.e., the region Voeren; cluster E2) and crossed the river Meuse. Starting from the 1990s, these clusters expanded towards each other, resulting in admixture at the contact zone, specifically in the zone between a motorway and canal (E313 and Albert canal, respectively). West of Brussels, population density and spatial extent of occurrence increased while two genetic clusters emerged (clusters W1 and W2). Although there was some exchange between the eastern and western groups, they remained largely genetically distinct. Similar to the reintroduced American martens ( *Martes americana* Turton, 1806) studied by Williams and Scribner (2010), the genetic clusters' location, size and membership strongly argued against genetic drift as the principal driver in the development of distinct genetic clusters. Our findings suggest that ancestry in combination with rapidly occurring genetic changes due to range expansion (Hagen et al. 2015) are major forces for the overall spatial structure. Still, drift has contributed to the divergence of cluster W2 and the lower genetic diversity found in cluster E1 (stronghold).

Consequently, the results suggest at least three different sources for the expansion, with the old stronghold as one of them. The geographical translation of a range of autopsy parameters relating to the age and reproductive status of dead specimens previously suggested that recolonisation occurred over a broad front rather than eccentrically from the stronghold (Van Den Berge et al. 2012). The stronghold, however, did not significantly expand to new areas. The lower and decreasing genetic diversity in the stronghold suggests minimal influx of migrants, possibly due to territory avoidance (priority effects) or other regional factors. As stone martens from cluster E2 moved towards the stronghold, territories on the north‐east side of cluster E1 became occupied, potentially causing stone martens from the stronghold to avoid these areas due to their territorial nature. This could suggest that the E2 lineage expanded more quickly than E1. In general, close relationships were found mostly within genetic clusters, with more related pairs (second cousins or higher) in the southeast, especially within the stronghold, indicating a tendency for philopatry. Whether the cause is actually behavioural or if the stronghold was not at carrying capacity and therefore did not deliver many dispersers is hard to unravel. Expansion from the stronghold towards the south is on the other hand a possibility which cannot be elucidated with our current sampling scheme. Also, local conditions, for example due to variation in how humans use and inhabit their properties, could influence the distribution of stone marten, as was found for Norway rat in New York (Johnson et al. 2016).

In the region west of Brussels, a northward colonisation occurred, as evidenced by decreased genetic diversity (sMLH) in that direction. Sporadic sightings until the start of recolonisation suggest a few stone martens may have survived the bottleneck, explaining the distinct genetic signature similar to that found in Fennoscandinavian wolverines despite current gene flow (Lansink et al. 2022). However, genetic diversity within western clusters was comparable to or higher than in the east, increasing over time, which indicates serial founder events and ongoing immigration (Hagen et al. 2015). Only a few dyads of moderate relatedness connected individuals from the eastern region with the west, suggesting source populations are more likely to be located south of western clusters, such as in the north of France (Hauts‐de‐France) and the west of the Walloon region where similar range expansions of stone marten were noted (Fournier 2000; Libois 1993, 1996, 2006).

In addition to conspecific avoidance playing a role in the expansion pattern, interspecific avoidance might also have been of influence. Since the pine marten was very rare in Flanders, especially during the study period (Van Den Berge et al. 2022), competition with this species was considered of little consequence. On the other hand, red foxes ( *Vulpes vulpes* , L. 1758) are an omnipresent, equally opportunistic species. Dietary analysis showed that while food resources for red foxes and stone martens overlap, their diet composition is different (Van Den Berge et al. 2022). Whether the expansion of the stone marten was partially dependent on the density of red foxes could be a further line of inquiry. According to a study by Zalewska et al. (2021), stone martens can adapt their use of space to reduce the risk of encountering larger carnivores.

### 4.2 Isolation‐By‐Distance Versus Landscape Effects

No difference between sexes in the spatial autocorrelation pattern was found, potentially due to the expansion with high immigration and recent exponential population growth causing density‐dependent dispersal (Bertrand et al. 2017; Bowler and Benton 2005; Carr et al. 2007; Dussex et al. 2016). Since stone martens are still establishing their territories during colonisation, the usual differences in home range sizes between males and females—and the resulting effects on genetic spatial patterns—may not yet be fully developed. The isolation‐by‐distance (IBD) models, using MLPE, were significant for every period investigated. The presence of IBD in such a mobile species is not uncommon, even at smaller regional scales (Vergara et al. 2015, 2017; Wereszczuk et al. 2017). A significant spatial pattern was also found when MEMs were used as explanatory variables in dbRDA models (results not shown) for the total dataset, each time period, each region (east and west of Brussels), and their combinations. The spatial pattern extended to a certain distance (i.e., the *x*‐intercept of the correlogram) from which individuals were no more related to each other than would be expected under random mating; this is the so‐called genetic patch. The genetic patch size seemed to increase over time, implying an increase in dispersal distances. Whether this increase is due to spatial assortment of dispersal ability at the expansion front, is pushed by high‐density populations, or a combination of both (Phillips et al. 2010) is unclear, and would need a wider sampling scheme and information on demography, physiological and morphological traits.

Heterogeneity in gene flow across the landscape was observed when accounting for isolation‐by‐distance (IBD), suggesting that genetic structuring is influenced by areas of reduced dispersal. Early stages of recolonisation showed higher gene flow within the stronghold, which advanced north over time and allowed exchange with the eastern cluster E2. Nonetheless, this exchange between lineages was limited to a small zone. In the western region, gene flow was less hindered, with potential barriers like motorways being crossed. Still, gene flow northwards was restricted, and high resistance between eastern and western populations became apparent. These areas with less gene flow than expected under IBD often coincided with the city of Brussels, motorways and canals. Although dbRDA results provide further evidence of the influence of urban elements such as motorways on gene flow, their effects were small and depended on the time frame and area under investigation. Variability in resistance posed by roads and waterways could be due to the number of accessible over‐ and underpasses which make crossing roads more feasible. Ascensão et al. (2014) found variability in usage of passages by stone marten. They assumed that non‐residents are unaware of locations for safe crossings. This supports our finding of no meaningful influence of motorways or other roads in the western region on inter‐individual genetic distance. This recently colonised area might hold relatively more individuals in the exploration phase than the region east of Brussels. As these individuals would be unfamiliar with the landscape, they would be more inclined to cross roads at various places and potentially cross roads more often than those found in the stronghold.

Geographic distance and landscape elements like tree cover density, navigable waterways, and grassland proportion explained genetic distances among individuals in the region west of Brussels. Limited influence of these factors suggests time might have been insufficient to find clear associations with landscape elements (Landguth et al. 2010). As such, rivers separating established populations of stone marten in Iberia appeared to partly explain population structure (Basto et al. 2016; Vergara et al. 2015). In the eastern region, reduced models explained more variance over time, highlighting the influence of motorways, tree cover density, and built‐up areas on genetic structure, beside the lingering effect of the ancestral population. Green areas with higher levels of tree cover density, such as parks and woodlands, remain attractive urban landscape features (Capon et al. 2021; Tóth et al. 2009), though large forests are not preferred by stone martens and could induce population isolation (Wereszczuk et al. 2017; Wereszczuk and Zalewski 2019). Regional differences in forest cover exist in Flanders. Except for a large forest complex south of Brussels, most forests consist of small fragments which could explain their limited influence in our models as opposed to urban land cover.

In contrast, motorways had a significant influence on the recolonisation of stone marten. Reduced models for both regions together showed higher adjusted *R* ^2^ at a larger radius (30 km), though with a higher value in the first period (4.9%) and similar, lower values for the two following periods (2.6%–2.9%), of which the largest amount was explained by motorways and to a degree by built‐up area, secondary road or waterway density. Although ancestry was not included in the total model, the MEMs captured this spatial structure. Multiple roads with heavy traffic connect Antwerp and Brussels, which coincides with the divide between east and west. Moreover, a canal connected to the river Scheldt and several larger cities can be found parallel to or along this axis. In the Bresse plains region in eastern France, buildings also appeared to potentially pose a hindrance to gene flow in stone marten (Larroque et al. 2016). The area was, however, significantly less urbanised, which reduces encounters of stone martens with residential areas. Still, impervious surfaces are less abundantly present in and around the stronghold; the area holds more open spaces, which could account for the lack of resistance to gene flow in that area.

As our sampling approach involved mostly traffic casualties, the influence of road mortality on the genetic structure found could be inflated. This sampling scheme can be considered non‐random, potentially targeting specific demographic groups (e.g., juveniles, individuals with higher movement tendencies or lower fitness). However, age estimates (based on cementum analysis and reproductive characteristics) indicated a mixture of ages, including individuals both in the exploration and settlement stage (unpublished data). In addition, the range of expansion seemed to increase over time, and closely related pairs were found over great distances that suggested crossing of those same roads. Although increased connectivity could come with increased road‐related mortality (Grilo et al. 2011), annual survival is probably high enough to mitigate the mortality risk posed by marten‐vehicle collisions (e.g., Crawford et al. 2018; Etter et al. 2002). Since the stone marten is crepuscular and nocturnal and therefore avoids daytime traffic, this seems plausible. Grilo et al. (2012) found that stone martens steered clear of motorways at times of high traffic intensity. Although roads are not a total barrier, they have been shown to negatively impact gene flow in mustelids (Garroway et al. 2011), including stone marten (Vergara et al. 2017).

### 4.3 Future Genetic Homogenisation

Eeraerts et al. (2022) found that the diet of the stone marten in the study area changed over time (the same period as in our study). Stone marten appeared to eat fewer fruits, grains, and nuts, and lagomorphs, while the proportion of edible household waste increased. Geographical differences were not investigated by the authors (e.g., the stronghold is located in a region rich with orchards of cultivated fruit). However, some behavioural shifts seemed to be at play. Whether or not the colonising stone marten is more tolerant to an urban environment remains to be investigated; it could be the reason behind the limited admixture between the old and the new. Still, barriers to gene flow and the relatively short time since secondary contact in our study might have contributed to the lack of homogenisation. In addition, spatial configuration rather than just the amount of habitat fragments and of potential barriers could be substantially different among regions, which impacts colonisation rate and success (Baguette and Van Dyck 2007). Gene flow among certain groups seemed to increase within the time scale of the study and included some long‐distance dispersals. At the same time, certain groups became more differentiated. This seems to contradict the homogenisation of the genetic clusters in the near future.

As the more traditional habitat of stone marten has become less available and its quality more variable across the landscape, the study of Eeraerts et al. (2022) could indicate that the species has become more dependent on anthropogenic resources. This might explain the current colonisation of highly urbanised areas. Whether dispersal from the stronghold is more costly due to local conditions (Johnson et al. 2009) or is heritable and habitat‐dependent (Saatoglu et al. 2024) could be further lines of inquiry. Miles et al. (2019) questioned the tendency to generalise the similar pressures and responses caused by urban environments, suggesting instead investigating how variation in traits among both species and populations responds to urbanisation. Understanding the factors behind recovery and expansion, and the variability in responses to urbanisation among populations will provide deeper insights into adaptability and genetic dynamics in urban landscapes of stone marten and species facing similar challenges.

## Author Contributions

**Karen Cox:** conceptualization (equal), data curation (lead), formal analysis (lead), investigation (lead), methodology (lead), resources (equal), validation (lead), visualization (lead), writing – original draft (lead), writing – review and editing (lead). **Jan Gouwy:** conceptualization (equal), data curation (supporting), investigation (supporting), resources (equal), writing – original draft (supporting), writing – review and editing (supporting). **Joachim Mergeay:** conceptualization (equal), methodology (supporting), writing – original draft (supporting), writing – review and editing (supporting). **Sabrina Neyrinck:** investigation (supporting), resources (equal), writing – original draft (supporting), writing – review and editing (supporting). **Koen Van Den Berge:** conceptualization (equal), data curation (supporting), investigation (supporting), resources (equal), writing – original draft (supporting), writing – review and editing (supporting).

## Ethics Statement

Road casualties of stone marten were collected according to regional regulations.

## Conflicts of Interest

The authors declare no conflicts of interest.

## Supporting information


Appendix S1.

## Data Availability

Raw sequence data for the GBS dataset are available in the European Nucleotide Archive (ENA) at EMBL‐EBI under the BioProject accession number PRJEB85085. The filtered SNP data set and accompanying sample information are accessible at Dryad Digital Repository: https://doi.org/10.5061/dryad.3xsj3txrn.
