# Supplementary material for: Rapid Colonisation of Synanthropic Stone Martens in a Highly Urbanised Region: Insights From Temporal and Spatial Analysis
Source: Ecol Evol. 2025 May 26;15(5):e71392. doi: 10.1002/ece3.71392 (PMC12104667; doi:10.1002/ece3.71392)
Supplement: Supplementary file 1 — Appendix S1. [file ECE3-15-e71392-s001.pdf]

Supplemental Information for:

Rapid colonisation of synanthropic stone martens in a highly urbanised region: Insights from temporal and spatial analysis

Karen Cox, Jan Gouy, Joachim Mergeay, Sabrina Neyrinck, Koen Van Den Berge

Table of Contents:

**Supplementary tables**	Page 2
**Supplementary figures**	Page 10

TABLE S1. Results of the isolation-by-distance models using a linear mixed model with maximum-likelihood population effects for each of three periods and the total dataset, separately. The sample in the Ardennes outside the main study area was not included. Significant *p*-values are indicated in bold.

**Period**	**estimate**	**SE**	**t-value**	***p***
1995-2002	0.00002	0.00000044	45.08407	**< 0.001**
2003-2007	0.00001	0.00000018	74.18213	**< 0.001**
2008-2013	0.00001	0.00000021	68.60172	**< 0.001**
total	0.00001	0.00000007	197.6835	**< 0.001**

TABLE S2. Pairwise F_{ST} (below diagonal) among genetic clusters of stone martens resulting from sNMF analyses for (a) 1995-2002, (b) 2003-2007, and (c) 2008-2013, with 95% confidence intervals (above diagonal). The names of the genetic clusters refer to those indicated in Figure 2.

(a)

1995-2002	W	E1	E2
W		0.053-0.096	0.045-0.092
E1	0.071		0.040-0.065
E2	0.066	0.051	

(b)

2003-2007	W1	W2	E1	E2
W1		0.037-0.071	0.071-0.094	0.060-0.086
W2	0.051		0.052-0.079	0.041-0.070
E1	0.081	0.063		0.032-0.050
E2	0.071	0.054	0.04	

(c)

2008-2013	W1	W2	E1	E2
W1		0.042-0.063	0.067-0.087	0.049-0.075
W2	0.051		0.061-0.078	0.047-0.072
E1	0.076	0.069		0.022-0.044
E2	0.061	0.059	0.031	

TABLE S3 Results of generalised least-squares models with individual standardised multilocus heterozygosity (sMLH) of stone marten as the response variable, and with longitude, latitude and their interaction as response variables. Models were created for the total dataset, for samples collected in the eastern part and for those collected in the western part of the study area (i.e. east and west of Brussels, respectively).

	**total**				**East**				**West**			
**variable**	**estim**	**SE**	**t-value**	***p***	**estim**	**SE**	**t-value**	***p***	**estim**	**SE**	**t-value**	***p***
intercept	0.997	0.0068	147.531	**< 0.0001**	0.977	0.0083	117.45	**< 0.0001**	1.039	0.012	86.875	**< 0.0001**
longitude	-0.023	0.0068	-3.442	**0.0006**	0.002	0.0088	0.2	0.8416	0.006	0.012	0.526	0.6001
latitude	-0.017	0.0068	-2.473	**0.0138**	-0.002	0.0084	-0.192	0.8479	-0.043	0.0119	-3.631	**0.0004**
longitude:latitude	0.017	0.0066	2.604	**0.0096**	-0.001	0.0085	-0.175	0.8616	0.009	0.0138	0.656	0.5132

Note: estim, estimate.

TABLE S4. Full models of the distance-based redundancy analysis (dbRDA) of genetic distances (Bray-Curtis distances) among stone martens and land cover properties conditioned on spatial structure (Moran Eigenvector Maps) for the total dataset, for the samples collected in het eastern part of the study area, and for the samples collected in the western part (i.e. east and west of Brussels, respectively).

set	period	radius (km)	full model conditional on MEMs	df	SS	F	*p*	R^2_{adj}
total	1995-2013	2	built-up + prim. roads + sec. roads + tree cover density + grassland + navigable waterways + motorways	20	0.1833	1.2142	**0.001**	0.0121
		5	built-up + prim. roads + sec. roads + tree cover density + grassland + navigable waterways + motorways	20	0.1826	1.2084	**0.001**	0.0118
		10	built-up + sec. roads + tree cover density + grassland + navigable waterways + motorways	19	0.1752	1.2206	**0.001**	0.0119
		30	built-up + grassland + navigable waterways + motorways	17	0.1592	1.2396	**0.001**	0.0115
	1995-2002	2	built-up + sec. roads + tree cover density + grassland + navigable waterways + motorways	16	0.1710	1.2660	**0.001**	0.0450
		5	built-up + sec. roads + tree cover density + grassland + navigable waterways + motorways	16	0.1764	1.3194	**0.001**	0.0534
		10	built-up + sec. roads + tree cover density + grassland + navigable waterways + motorways	16	0.1748	1.3037	**0.001**	0.0509
		30	built-up + sec. roads + tree cover density + grassland + navigable waterways + motorways	16	0.1783	1.3388	**0.001**	0.0565
	2003-2007	2	built-up + sec. roads + tree cover density + grassland + navigable waterways + motorways	18	0.1734	1.1919	**0.001**	0.0247
		5	built-up + sec. roads + tree cover density + grassland + navigable waterways + motorways	18	0.1726	1.1851	**0.001**	0.0238
		10	built-up + sec. roads + tree cover density + grassland + navigable waterways + motorways	18	0.1760	1.2134	**0.001**	0.0274
		30	built-up + prim. roads + sec. roads + tree cover density + grassland + navigable waterways + motorways	19	0.1844	1.2053	**0.001**	0.0278
	2008-2013	2	built-up + prim. roads + sec. roads + tree cover density + grassland + navigable waterways + motorways	18	0.1806	1.1742	**0.001**	0.0251
		5	built-up + prim. roads + sec. roads + tree cover density + grassland + navigable waterways + motorways	18	0.1801	1.1707	**0.001**	0.0246
		10	built-up + prim. roads + sec. roads + tree cover density + grassland + navigable waterways + motorways	18	0.1835	1.1986	**0.001**	0.0285
		30	built-up + sec. roads + tree cover density + grassland + navigable waterways + motorways	17	0.1772	1.2285	**0.001**	0.0309
east	1995-2013	2	built-up + sec. roads + tree cover density + grassland + navigable waterways + motorways + ancestry	13	0.1451	1.5200	**0.001**	0.0306
		5	built-up + sec. roads + tree cover density + grassland + navigable waterways + motorways + ancestry	13	0.1453	1.5215	**0.001**	0.0307
		10	built-up + sec. roads + tree cover density + grassland + navigable waterways + motorways + ancestry	13	0.1464	1.5350	**0.001**	0.0315
		30	built-up + tree cover density + grassland + motorways + ancestry	11	0.1285	1.5888	**0.001**	0.0294
	1995-2002	2	built-up + sec. roads + tree cover density + grassland + navigable waterways + motorways + ancestry	12	0.1160	1.1166	**0.026**	0.0278

set	period	radius (km)	full model conditional on MEMs	df	SS	F	*p*	R^2_{adj}
		5	built-up + sec. roads + tree cover density + grassland + navigable waterways + motorways + ancestry	12	0.1171	1.1303	**0.019**	0.0309
		10	built-up + sec. roads + tree cover density + grassland + navigable waterways + motorways + ancestry	12	0.1159	1.1145	**0.028**	0.0273
		30	built-up + sec. roads + tree cover density + grassland + motorways + ancestry	11	0.1048	1.0904	0.083	0.0199
	2003-2007	2	built-up + sec. roads + tree cover density + grassland + navigable waterways + motorways + ancestry	13	0.1249	1.2406	**0.001**	0.0370
		5	built-up + sec. roads + tree cover density + grassland + navigable waterways + motorways + ancestry	13	0.1262	1.2564	**0.001**	0.0394
		10	built-up + sec. roads + tree cover density + grassland + navigable waterways + motorways + ancestry	13	0.1268	1.2641	**0.001**	0.0405
		30	built-up + tree cover density + grassland + motorways + ancestry	11	0.1088	1.2755	**0.001**	0.0359
	2008-2013	2	built-up + sec. roads + tree cover density + grassland + navigable waterways + motorways + ancestry	12	0.1183	1.2545	**0.001**	0.0575
		5	built-up + sec. roads + tree cover density + navigable waterways + motorways + ancestry	11	0.1098	1.2673	**0.001**	0.0555
		10	built-up + sec. roads + tree cover density + motorways + ancestry	10	0.1019	1.2938	**0.001**	0.0554
		30	built-up + motorways + ancestry	8	0.0823	1.2905	**0.001**	0.0444
west	1995-2013	2	built-up + prim. roads + sec. roads + tree cover density + grassland + navigable waterways + motorways	12	0.1074	1.0571	0.159	0.0096
		5	built-up + prim. roads + sec. roads + tree cover density + grassland + navigable waterways + motorways	12	0.1086	1.0727	0.115	0.0122
		10	built-up + sec. roads + tree cover density + grassland + navigable waterways + motorways	11	0.1018	1.1001	**0.048**	0.0153
		30	built-up + grassland + navigable waterways + motorways	9	0.0831	1.0930	0.076	0.0117
	2003-2007	2	built-up + prim. roads + sec. roads + tree cover density + grassland + navigable waterways + motorways	11	0.0992	0.9623	0.604	-0.0174
		5	built-up + prim. roads + sec. roads + tree cover density + grassland + navigable waterways + motorways	11	0.0996	0.9694	0.628	-0.0141
		10	built-up + sec. roads + grassland + navigable waterways + motorways	9	0.0789	0.9230	0.777	-0.0294
		30	built-up + grassland + navigable waterways + motorways	8	0.0700	0.9260	0.756	-0.0251
	2008-2013	2	built-up + prim. roads + sec. roads + tree cover density + grassland + navigable waterways + motorways	11	0.1147	1.1511	**0.041**	0.0402
		5	built-up + prim. roads + sec. roads + tree cover density + grassland + navigable waterways + motorways	11	0.1131	1.1276	0.060	0.0342
		10	built-up + prim. roads + sec. roads + tree cover density + grassland + navigable waterways + motorways	11	0.1175	1.1949	**0.014**	0.0512
		30	built-up + grassland + navigable waterways + motorways	8	0.0855	1.1708	**0.031**	0.0333

Note: Set indicates the dataset used for the analysis. The radius represents the buffer radius used to calculate the proportion of different land cover classes surrounding the sampling locations and total length of navigable waterways, primary and secondary roads. Motorways were included as a categorical (or dummy) variable. R^2_{adj} is the adjusted R^2 for the entire model. Significant *p*-values are indicated in bold.

TABLE S5. Results of the distance-based redundancy analysis (dbRDA) of genetic distances (Bray-Curtis distances) between stone martens and land cover properties conditioned on spatial structure (Moran Eigenvector Maps) for different time periods, including the total dataset or samples collected in het eastern and western part of the study area (i.e., east and west of Brussels, respectively).

set	period	radius (km)	model R^2_{adj}	variable	df	SS	F	p_{term}	$p_{marginal}$	R^2_{adj}
total	1995-2002	2 and 5	0.0442	motorways	11	0.1283	1.3801	**0.001**	**0.001**	0.0442
		10	0.0474	motorways	11	0.1283	1.3854	**0.001**	**0.001**	0.0399
				tree cover density	1	0.0107	1.2667	**0.015**	**0.023**	0.0033
		30	0.0491	motorways	11	0.1283	1.3881	**0.001**	**0.001**	0.0400
				built-up	1	0.0118	1.3998	**0.004**	**0.003**	0.0049
	2003-2007	2 and 5	0.0218	motorways	13	0.1301	1.2341	**0.001**	**0.001**	0.0218
		10	0.0236	motorways	13	0.1301	1.2369	**0.001**	**0.001**	0.0218
				navigable waterways	1	0.0099	1.2217	**0.013**	**0.023**	0.0018
		30	0.0264	motorways	13	0.1301	1.2412	**0.001**	**0.001**	0.0193
				navigable waterways	1	0.0109	1.3462	**0.004**	**0.001**	0.0032
				built-up	1	0.0099	1.2232	**0.026**	**0.027**	0.0018
	2008-2013	2	0.0225	motorways	12	0.1256	1.221	**0.001**	**0.001**	0.0215
				sec. roads	1	0.0103	1.1955	**0.038**	**0.050**	0.0018
		5	0.0207	motorways	12	0.1252	1.2145	**0.001**	**0.001**	0.0207
		10	0.0236	motorways	12	0.1252	1.2187	**0.001**	**0.001**	0.0215
				sec. roads	1	0.0113	1.3140	**0.013**	**0.009**	0.0029
		30	0.0290	motorways	12	0.1252	1.2268	**0.001**	**0.001**	0.0217
				sec. roads	1	0.0130	1.5276	**0.001**	**0.001**	0.0051
				built-up	1	0.0118	1.3861	**0.006**	**0.001**	0.0035
east	1995-2002	2, 5 and 30	0.0172	ancestry	1	0.0163	1.8561	**0.001**	**0.001**	0.0172
		10	0.0237	ancestry	1	0.0163	1.8694	**0.001**	**0.001**	0.0183
				sec. roads	1	0.0115	1.3162	**0.035**	**0.047**	0.0065
	2003-2007	all	0.0324	motorways	7	0.0698	1.2801	**0.001**	**0.001**	0.0211
				ancestry	1	0.0137	1.7581	**0.001**	**0.001**	0.0100
	2008-2013	2	0.0480	motorways	6	0.0614	1.2895	**0.001**	**0.001**	0.0290
				ancestry	1	0.0117	1.4769	**0.001**	**0.009**	0.0076
				grassland	1	0.0104	1.2928	**0.013**	**0.024**	0.0070
		5 and 30	0.0411	motorways	6	0.0614	1.2795	**0.001**	**0.001**	0.0267
				ancestry	1	0.0117	1.4655	**0.001**	**0.002**	0.0102
		10	0.0544	motorways	6	0.0614	1.2987	**0.001**	**0.001**	0.0254
				ancestry	1	0.0117	1.4875	**0.002**	**0.005**	0.0095
				built-up	1	0.0103	1.3098	**0.011**	**0.006**	0.0083
				sec. roads	1	0.0102	1.2893	**0.012**	**0.018**	0.0066
west	2008-2013	2	0.0121	prim. roads	1	0.0139	1.4833	**0.018**	**0.026**	0.0121
		10	0.0113	tree cover density	1	0.0136	1.4532	**0.021**	**0.026**	0.0113

Note: Set indicates the dataset used for the analysis. The radius represents the buffer radius used to calculate the proportion of different land cover classes surrounding the sampling locations and total distances of navigable

waterways, primary and secondary roads. Motorways were included as as a categorical (or dummy) variable. Model R^2_{adj} is the adjusted R^2 for the entire model, while the last column shows the value for each variable after variation partioning. df, degrees of freedom; SS, sum of squares; F, F-statistic; p_{term}, p-value per term (sequentially from first to last); $p_{marginal}$, the p-value of the marginal effect of the terms (each marginal term analysed in a model with all other variables). Significant p-values are indicated in bold.

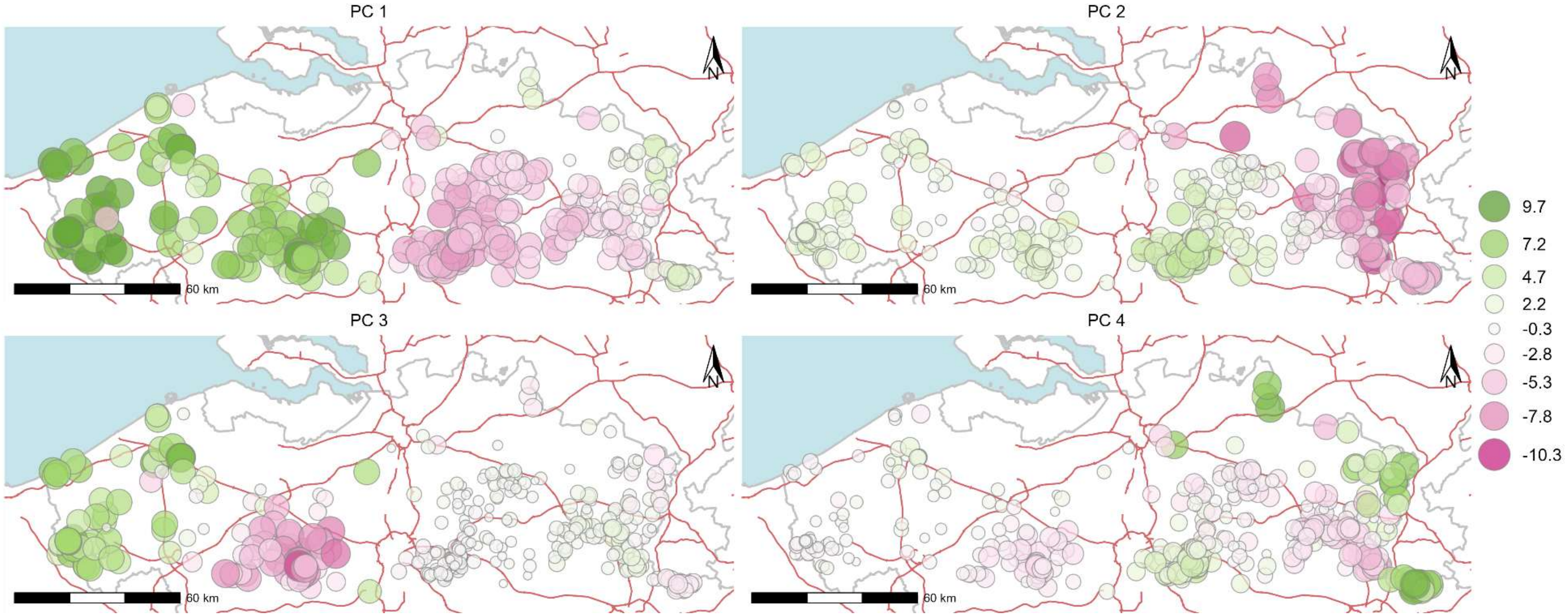

FIGURE S1

First four principal components plotted as circles with size and colour scaled relative to the values, resulting from a principal component analysis of the total dataset of stone marten genotypes in Flanders. Country borders are indicated with grey lines. Major motorways are shown as red lines.

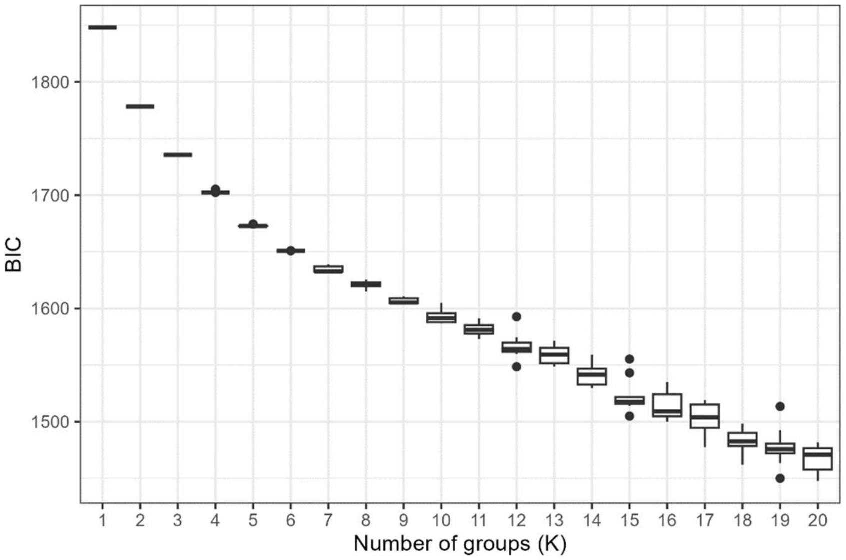

FIGURE S2
Bayesian Information Criterion (BIC) values for all 20 groups (K) evaluated with the K-means procedure. Boxplots of BIC for each K were constructed based on ten replicates.

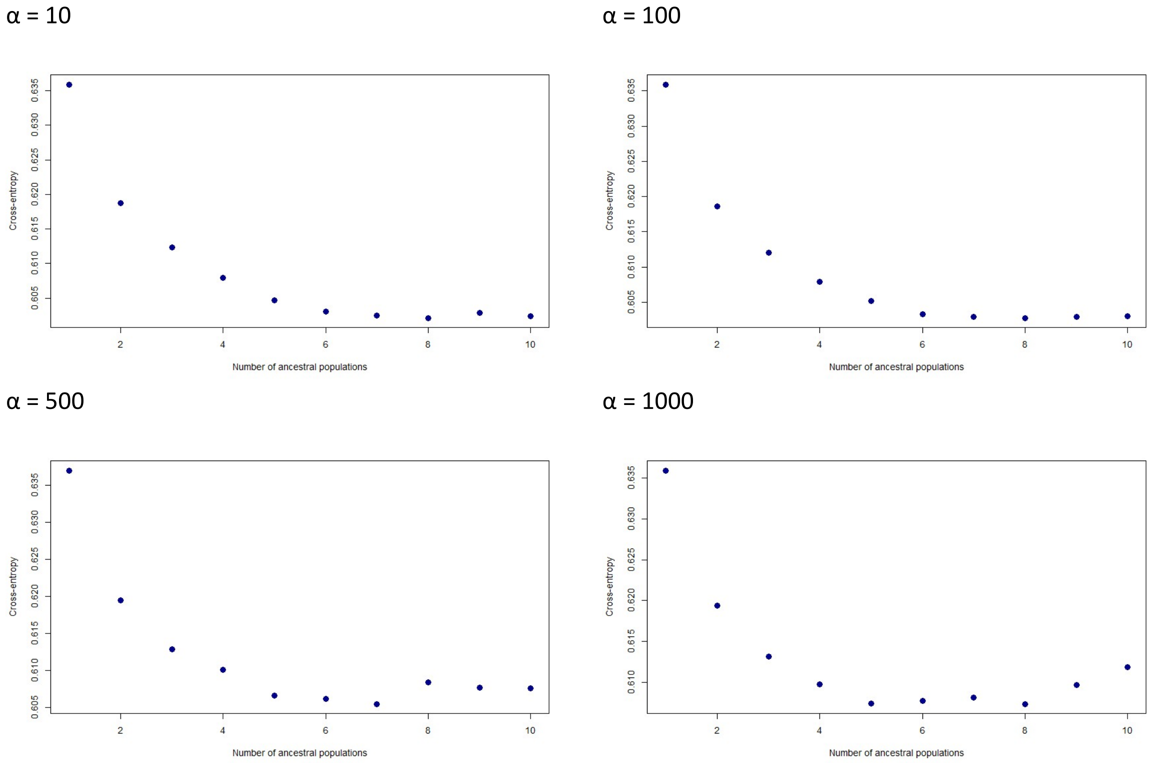

FIGURE S3
Minimum cross-entropy values obtained for one to ten ancestral populations using different regularisation parameters (α) and ten replicates for each number of populations.

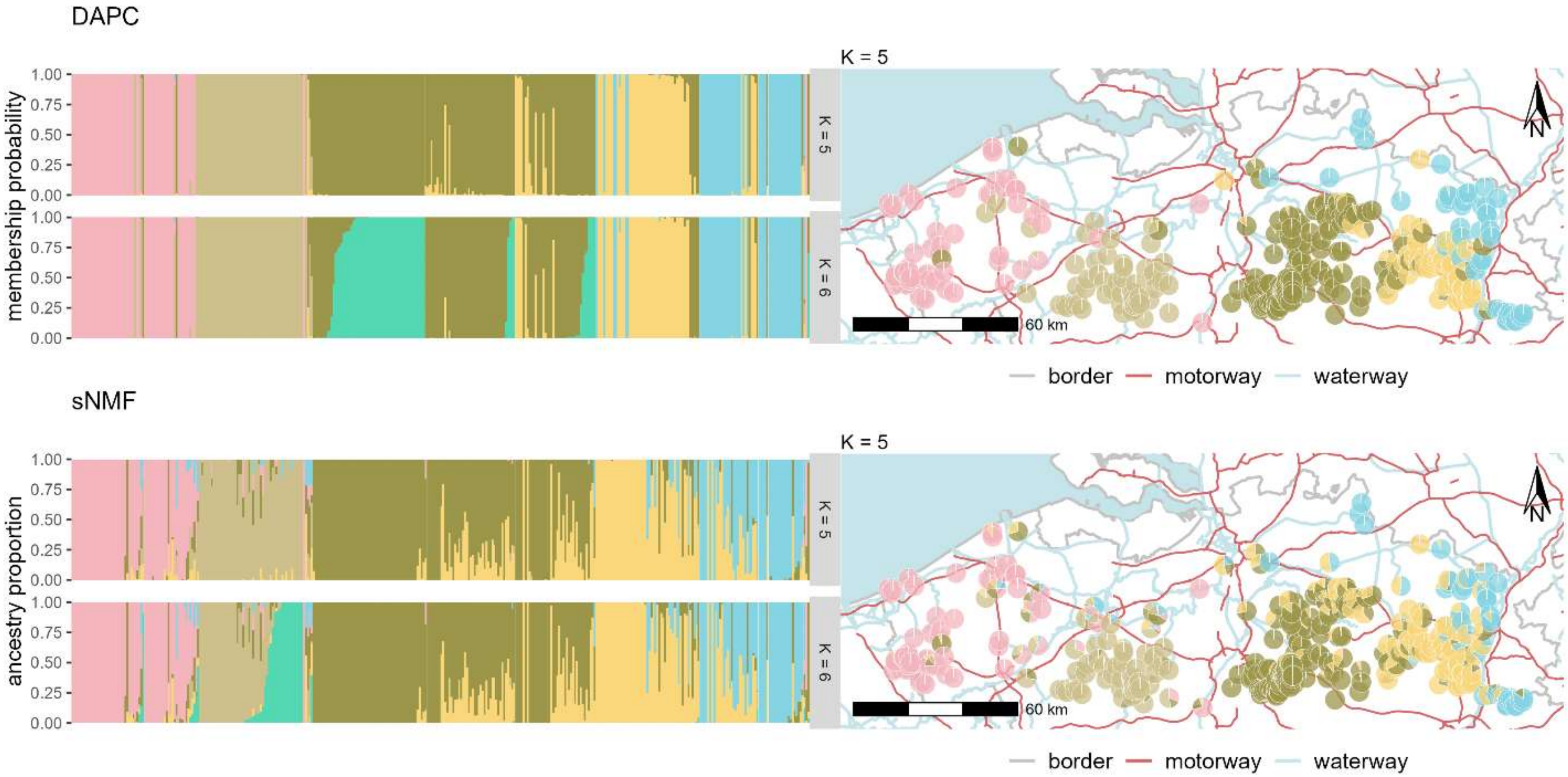

FIGURE S4

Results using Discriminant Analysis of Principal Components (DAPC; upper panel) and sparse Non-Negative Matrix Factorization algorithms (sNMF; lower panel). Cluster memberships are shown as barplots for 5 and 6 clusters (left), and as pie charts for 5 clusters on a map of Flanders (right) with a different colour for each cluster.

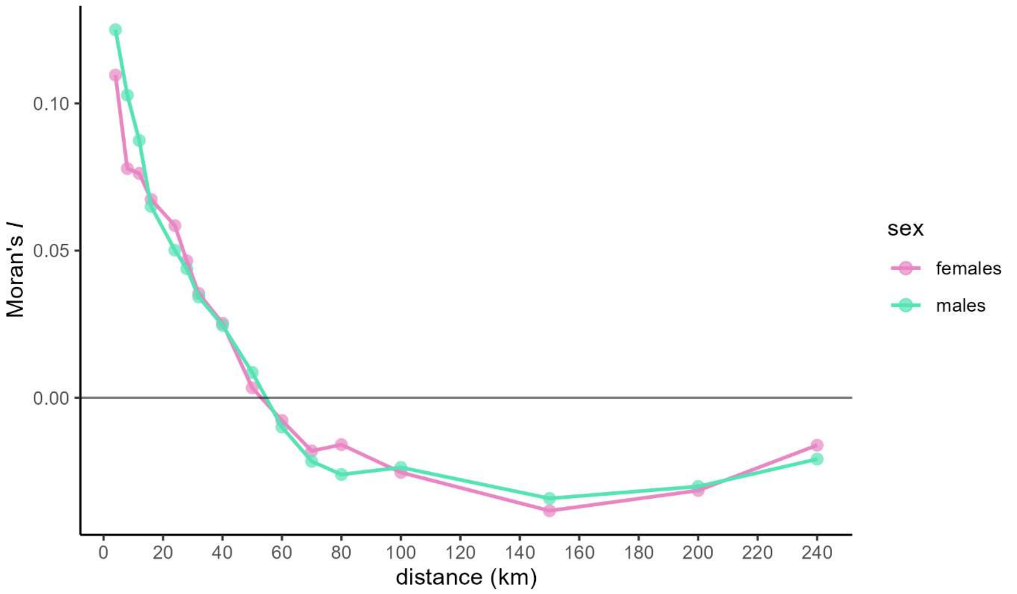

FIGURE S5

Moran's *I* correlogram for pairs of female (pink) and male (green) stone martens, respectively, across 16 distance intervals. Each point represents the mean pair-wise Moran's *I* with significant spatial autocorrelation for the given distance class.

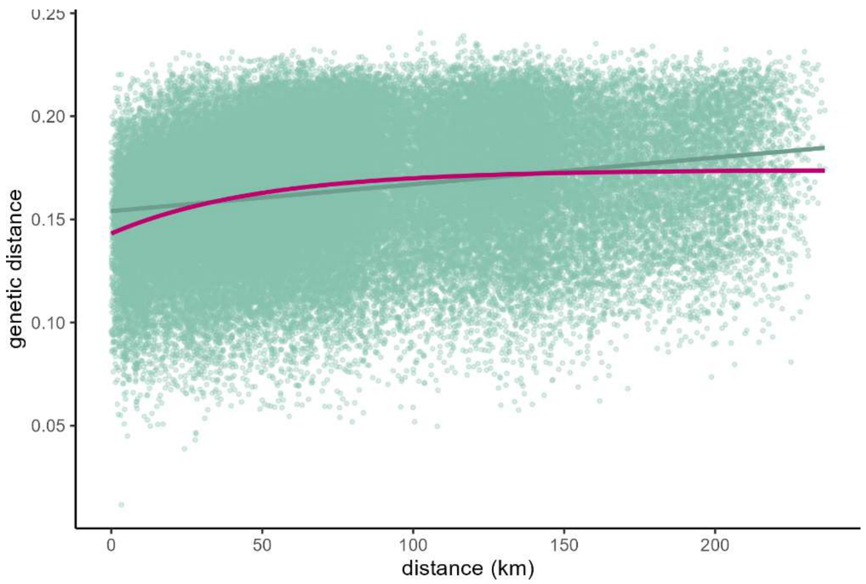

FIGURE S6

Isolation-by-distance plot of pairwise Euclidean geographic distances and Prevosti's genetic distances between sampled stone martens, including a fitted linear (green) and non-linear (pink) regression.

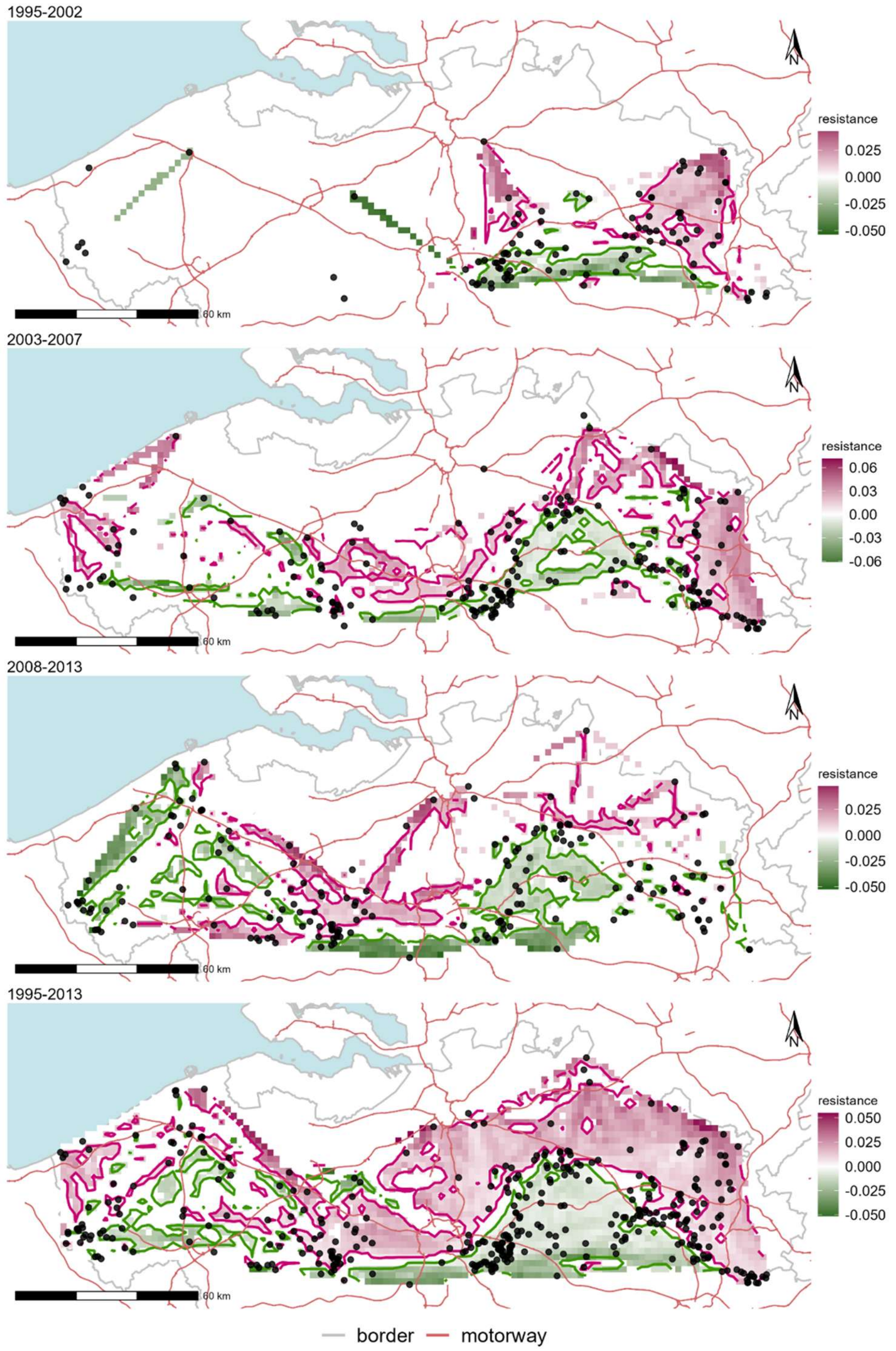

FIGURE S7

Results of IBD residual-based resistance mapping of stone marten in Flanders using Prevosti's genetic distance and non-linear trend, for each of three periods and the total dataset. The map consists of areas of statistically high (pink) and low (green) resistance to gene flow. Pink and green contour lines delineate areas with statistically significant high or low resistance, respectively, Blank cells are those with no statistical certainty, and black dots are sampling locations.
